# High-Resolution Structural Proteomics of Mitochondria Using the ‘Build and Retrieve’ Methodology

**DOI:** 10.1016/j.mcpro.2023.100666

**Published:** 2023-10-14

**Authors:** Zhemin Zhang, Marios L. Tringides, Christopher E. Morgan, Masaru Miyagi, Jason A. Mears, Charles L. Hoppel, Edward W. Yu

**Affiliations:** Department of Pharmacology, Case Western Reserve University School of Medicine, Cleveland, Ohio, USA

**Keywords:** dehydrogenase, isomerase, aminotransferase, dismutase, catalase

## Abstract

The application of integrated systems biology to the field of structural biology is a promising new direction, although it is still in the infant stages of development. Here we report the use of single particle cryo-EM to identify multiple proteins from three enriched heterogeneous fractions prepared from human liver mitochondrial lysate. We simultaneously identify and solve high-resolution structures of nine essential mitochondrial enzymes with key metabolic functions, including fatty acid catabolism, reactive oxidative species clearance, and amino acid metabolism. Our methodology also identified multiple distinct members of the acyl-CoA dehydrogenase family. This work highlights the potential of cryo-EM to explore tissue proteomics at the atomic level.

## Highlight

• Using cryo-EM to define the composition of a human mitochondrial lysate.
• Simultaneously identifying and solving structures of nine mitochondrial enzymes.
• Application of cryo-EM in studying high-resolution structural omics of human enzymes.

## Graphical Abstract

**Figure:**
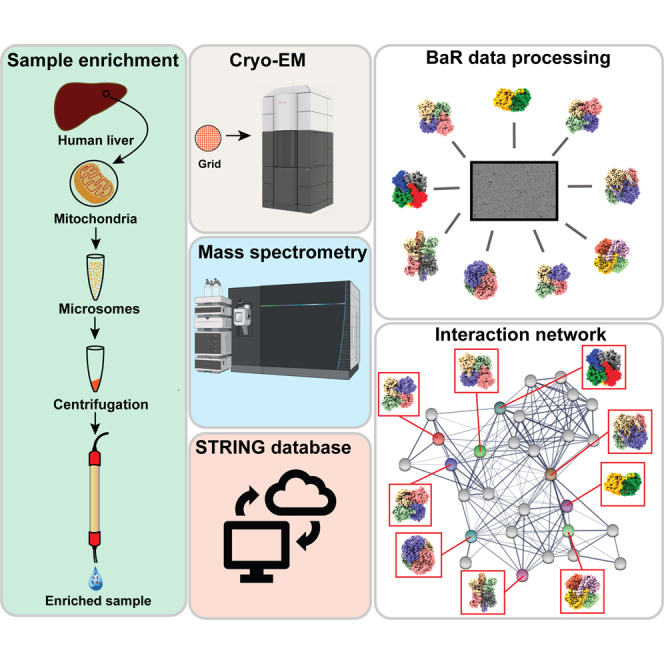

## In Brief

Zhang *et al* use cryo-EM to simultaneously identify and solve structures of nine different enzymes from human liver mitochondrial lysate. Their work highlights the potential of this methodology in facilitating structural proteomics of a biological system at high resolution.

Mitochondria are double membraned organelles essential for a wide variety of functions. The most prominent role of mitochondria is the production of ATP, the major energy source of the cell (1). ATP is produced though a multicomponent process called oxidative phosphorylation. Mitochondria are also involved in many other important cellular functions, including lipid metabolism, synthesis of iron-sulfur clusters, stress responses, and mediating cellular division and death (1, 2, 3, 4, 5, 6). At a population level, multiple mitochondria interact to form networks that are essential for proper cellular function. Given the ubiquitous role of mitochondria, dysfunction in these organelles can result in pathologies throughout the entire body. As a result, mitochondrial dysfunction is prevalent within genetically inherited metabolic disorders (1, 2, 3, 7).

Recent advances in mass spectrometry have vastly expanded the large scale analysis of proteomes of human tissues (8). The continuous development in “omics” technologies has expanded the scope and reliability of proteomics and systems biology (8). This systems proteomics approach has also dramatically enhanced our knowledge of mitochondrial biology. Currently, the list of mammalian mitochondrial proteins is quite comprehensive, allowing us to work towards a comprehensive understanding of this organelle at the structure/function level. Briefly, the mitochondrial proteome is coded by two genomes; mitochondrial and nuclear (7). Across the roughly 1500 distinct mitochondrial proteins, only 13 proteins are encoded by the human mitochondrial genome (7). Therefore, approximately 99% of them are encoded by nuclear genes and are imported into the mitochondria. These genes are differentially expressed in different tissues and cell types, with about half of the mitochondrial proteins being shared across all tissues and the other half tissue-specific (6, 7).

Recently, advancements in single-particle cryo-EM have facilitated the structural determination of traditionally challenging protein targets. Unlike mass spectrometry and proteomics, these structural tools typically require homogeneous and pure protein samples. This limits the ability to complement protein structure determination with existing systems biology approaches and explore native, heterogeneous samples. To address this challenge, our lab recently designed an approach to use cryo-EM to study tissue samples, called “Build and Retrieve” (BaR) (9). Specifically, BaR is an iterative methodology that relies on *in silico* purification. This methodology involves sorting images from a large and heterogeneous data set into different protein classes. The BaR platform includes preliminary 2D and 3D classifications and initial map constructions. Particle subsets with shared structural features are combined in order to create 3D *ab initio* templates. These initial templates are then used to extract additional particle views that share structural features, allowing for an expansion of the number of views in each protein class. The updated particle stacks are then processed using multiple rounds of 2D and 3D classifications in order to build high resolution maps for reviewing protein identities and solving final structural models. BaR is able to handle raw biological samples consisting of multiple proteins and enzymes, permitting us to extend the capacity of the structural tool of cryo-EM to study human tissues and organs in the context of systems biology.

To highlight the potential of the BaR methodology towards simultaneously elucidating structural information of a variety of mitochondrial proteins at near atomic resolutions, we enriched the protein component from human mitochondria lysate and then individually loaded the enriched protein samples onto cryo-EM grids for single particle imaging. Using this approach, we were able to simultaneously identify and solve cryo-EM structures of nine different human mitochondrial enzymes: short chain acyl-CoA dehydrogenase (SCAD), medium chain acyl-CoA dehydrogenase (MCAD), isovaleryl-CoA dehydrogenase (IVD), delta (3, 5)-delta (2, 4) dienoyl-CoA isomerase (ECH1), aspartate aminotransferase (GOT2), glutamate dehydrogenase (GLUD1), mitochondrial superoxide dismutase 2 (SOD2), catalase (CAT), and mitochondrial aldehyde dehydrogenase (ALDH2). Functionally, the three acyl-CoA dehydrogenases—SCAD, MCAD and IVD—are involved in beta oxidation and amino acid catabolism. The ECH1 isomerase is a key enzyme in an auxiliary beta oxidation pathway for odd numbered unsaturated fatty acids. GOT2 facilitates recycling of NADH *via* the interconversion of aspartate and α-ketoglutarate to oxaloacetate and glutamate. Glutamate metabolism is regulated by GLUD1. Finally, SOD2, CAT, and ALDH2 are all involved in clearing toxic byproducts of oxidative respiration.

## Experimental Procedures

### Human Mitochondrial Lysate

Mitochondria isolated from liver tissue were purchased from Xenotech (BioIVT). The mitochondria were resuspended in a buffer containing 20 mM Tris–HCl (pH 7.5), 100 mM NaCl, and 5 mM Na-cholate. The soluble lysate fraction was separated from the membrane fraction by ultracentrifugation at 20,000*g*. The extracted soluble lysate was then passed through a 0.22-μM filter and enriched using a Superdex 200 column (GE Healthcare) equilibrated with 20 mM Tris–HCl (pH 7.5) and 100 mM NaCl. We isolated three peaks based on the column separation. The first peak contained proteins ranging from 200 to 450 kDa including the identified ECH1 and GLUD1. Peak 2 contained proteins ranging in size from 150 to 250 kDa including the identified MCAD, IVD, SCAD, CAT, and ALDH2. Peaks 1 and 2 exhibited a degree of overlap. Our BaR methodology also identified CAT and ALDH2 subclasses in peak 1 but the particle stacks from peak 2 resulted in higher resolution maps. Lastly, peak 3 contained proteins smaller than 150 kDa including SOD2 and GOT2.

### Cryo-EM Sample Preparation

Protein sizes corresponding to each peak were used for cryo-EM analysis. 3.5 μl (0.07–0.08 mg/ml) of each sample was applied to Quantifoil R 2/2 Cu 200 holey grids coated with graphene oxide. Samples were blotted for 8 s, then plunge frozen into liquid ethane using a Vitrobot (Thermo Fisher). The resulting grids were stored in liquid nitrogen until data collection.

### Data Collection

Samples from peak 1, peak 2, and peak 3 were collected at Pacific Northwest Cryo-EM Center using a Titan Krios cryo-electron transmission microscope (Thermo Fisher Scientific). Images were recorded using 1.0 to 2.5 μM defocus on a K3 direct electron detector (Gatan) using super resolution at 105,000× magnification. The super resolution size was 0.4125 Å/px resulting in a physical pixel size 0.825 Å/px. A second set of peak 3 samples was collected at the Cleveland Center for Membrane & Structural Biology using a Titan Krios cryo-electron transmission microscope (Thermo Fisher Scientific). Images were recorded using 1.0 to 2.5 μM defocus on a K3 direct electron detector (Gatan) using super resolution at 81,000× magnification. The super resolution size was 0.535 Å/px resulting in a physical pixel size 1.07 Å/px.

### Data Processing

Initial image stacks were binned by a factor of 2 and motion corrected using cryoSPARC v3 (10). The patchCTF function was used to estimate the contrast transfer function (10). The Topaz tool, default ResNet16 (64 U) pre-trained model, was used to pick initial particle sets (11). The BaR protocol was used to separate different initial structure classes (9). First, during the “build” phase, particles were sorted *via* multiple rounds of 2D classification to identify particles with structural features. These particle stacks were then refined and grouped together to form initial 3D *ab initio* models. Initial models were built using C1 symmetry to reduce model bias. These models were then used as structural filters in a series of heterogeneous refinements. Each model “retrieves” additional particles relative to the initial stack, including a more diverse set of orientations. Refined particle stacks were then cleaned using 2D classification and used to generate refined 3D models. Non-uniform refinement was used to build the final maps. Once the high-resolution maps were built, the protein sequences were revealed using Deep Tracer (12). The resulting protein sequences were then used for sequence alignment in BLASTP (13), utilizing *Homo sapiens* as the target species to uncover the identities of these proteins. LC-MS/MS was also used to confirm the presence of these proteins in the samples that were used.

### Model Building and Refinement

Model building for all proteins was performed using Coot (14). The PHENIX suite program, phenix.real_space_refine (15), was used for structural refinements. Final atomic models were evaluated using MolProbity (16).

### Interaction Network

The interaction network connecting the nine proteins (SCAD, MCAD, IVD, ECH1, GOT2, GLUD1, SOD2, CAT, and ALDH2) was predicted using the STRING database v.11.5 (17). The enzymes from *H. sapiens* were input as a multiprotein search. The confidence score cutoff was set at 0.4. Interaction predictions were mainly derived from textmining, experiments, databases, co-expression, neighborhood, gene fusion, and co-occurrence, and other settings were set as default. The network contained 29 nodes and 171 edges. The average local clustering coefficient was 0.809, and average node degree was 16.9 with a protein-protein interaction enrichment *p*-value <1.0e^−16^. Line thickness was used to depict interaction confidence.

### Proteomic Analysis

For each peak from the enrichment process using size-exclusion chromatography, 4 μg of sample was separately denatured in a buffer containing 50 mM NH_4_HCO_3_ and 8 M urea. DTT was added to a final concentration of 10 mM at 25 °C for 30 min in order to reduce the sample. Alkylation was achieved using 25 mM iodoacetamide at 25 °C for 30 min. Samples were diluted by a factor of four using a digestion buffer containing 100 mM NH_4_HCO_3_ and trypsin/Lys-C mix (1:20 enzyme:substrate). Digestion took place overnight at 25 °C. The next day, digested peptides were desalted using a reverse-phase C18 Microspin column (Nest Group), washed two times with 150 μl aqueous solution containing 0.1% formic acid, and then eluted with 150 μl aqueous solution containing 80% acetonitrile and 0.1% formic acid.

LC-MS/MS was performed using a Thermo Scientific Fusion Lumos mass spectrometry system (Thermo Fisher Scientific). A Dionex 15 cm × 75 μm id Acclaim PepMap C18, 2 μm, 100 A reversed-phase capillary chromatography column was loaded with peptide. A linear gradient of acetonitrile (2–35%) in aqueous solution containing 0.1% formic acid was used for peptide chromatography for 90 min at a rate of 300 nl/min. The subsequent elution was introduced into the mass spectrometer operating in data-dependent MS to MS/MS switching mode with collision-induced dissociation mode. Full MS scanning was performed at 70,000 resolution between m/z = 350 and m/z = 1500. Protein identification was done by comparing experimental peptide MS/MS spectra to the UniProt human proteome database (Release-2022_04, downloaded on Feb 8th, 2022, total number of human entries = 79,038) using MaxQuant version 1.6.3.3 (18, 19). While searching the database, carbamidomethylation was added as static modifications, and methionine oxidation and N-terminal acetylation were added as variable modifications. The precursor ion tolerance was set to 4.5 ppm. The product ion tolerance was 0.5 Da. For peptide/protein identification, a strict trypsin specificity was applied: the minimum peptide length was set to 7, the maximum missed cleavage was set to 2, and the cutoff false discovery rate was set to 0.025.

### Experimental Design and Statistical Rationale

To confirm the presence of enzymes identified from BaR, we used *in vitro* proteomics approach to process the exact same samples used for single-particle cryo-EM data collection. Therefore, the peak 1, peak 2, and peak 3 mitochondrial lysate enriched from the Superdex 200 column (GE Healthcare) were individually aliquoted (2.0 μl from each lysate peak) and subjected to LC-MS/MS analysis (one biological repeat for each peak). The signal intensity, protein sequence coverage, and protein accession number of the top 10 to 15 identified proteins of each sample are listed in Supplemental Table S2. All proteomics data sets have been made available to the public by depositing them in ProteomeXchange *via* the PRIDE database.

## Results

Enrichment of the human liver mitochondrial lysate using size-exclusion chromatography resulted in three major protein peaks. The peak sizes corresponded to roughly 200 to 450 kDa, 150 to 250 kDa, and 80 to 150 kDa. We collected single particle cryo-EM images of each of the three peaks and processed them using the BaR methodology (Supplemental Fig. S1). The BaR approach yielded nine enzyme structures with resolutions ranging between 2.30 Å and 3.15 Å (Supplemental Table S1). These enzymes were identified as SCAD, MCAD, IVD, ECH1, GOT2, GLUD1, SOD2, CAT, and ALDH2. In order to validate the presence of these enzymes in our sample, we also analyzed the composition of each of the three peaks using proteomic analysis. The data indicated that each peak contained more than 400 proteins and the presence of each of our target proteins were identified in the corresponding peak (Supplemental Table S2).

To identify whether these nine human mitochondrial enzymes interact and cooperate with each other, we constructed an interconnected network using the STRING database (17). We postulate that these nine enzymes are involved in a range of interactions in the network (Supplemental Fig. S2).

### Acyl-coenzyme a Dehydrogenases

Acyl-coenzyme A dehydrogenases are a family of enzymes involved in fatty acid oxidation and amino acid catabolism (20, 21). Currently, eleven members of the ACAD family have been described in the human genome: SCAD, MCAD, long chain acyl-CoA dehydrogenase, very long chain acyl-CoA dehydrogenase, ACAD9, IVD, short/branched chain acyl-CoA dehydrogenase, isobutyryl-CoA dehydrogenase, glutaryl-CoA dehydrogenase, ACAD10, and ACAD11 (20, 21). Five of these—SCAD, MCAD, long chain acyl-CoA dehydrogenase, very long chain acyl-CoA dehydrogenase and ACAD9—are involved in the beta oxidation of fatty acids with different specificity for short, medium, or long chain fatty acid acyl-CoA substrates (21). Four others are involved in the amino acid catabolism of isoleucine (short/branched chain acyl-CoA dehydrogenase), leucine (IVD), lysine/tryptophan (glutaryl-CoA dehydrogenase), and valine (isobutyryl-CoA dehydrogenase) while the remaining two members (ACAD10 and ACAD11) have unknown functions (21). Amino acid sequence similarity ranges from 30 to 46% across the members of the family (21). The nine enzymes with identified function share common catalytic mechanisms with one flavin adenine dinucleotide (FAD) cofactor required per monomer.

Initially, our BaR approach generated one structural class representative of the acyl-coenzyme A family. By layering an additional 3D classification step (22) into the pipeline, we were able to resolve distinct high-resolution structures: (1) SCAD with cofactor and substrate, (2) MCAD with cofactor, and (3) IVD with cofactor (Supplemental Fig. S3). Importantly, our method was able to extract three unique but highly structurally related enzymes from a single heterogeneous sample. This work highlights an additional tantalizing potential of the BaR methodology in extracting multiple high-resolution structures among related proteins.

#### Short chain acyl-CoA dehydrogenase

Butanoyl-CoA and other four or five carbon fatty acid acyl-CoA molecules are the initial substrates of SCAD (23, 24, 25). Mutations in the corresponding *SCAD* gene can lead to SCAD deficiency (SCADD), a rare autosomal recessive disorder (26, 27). Many SCADD symptoms such as hypoglycemia, seizures, and myopathy are consistent with other acyl-CoA dehydrogenase deficiencies (26). In addition, developmental delay and other neurological symptoms are commonly seen in SCADD patients (26). These neurological symptoms are less frequently described in other β-oxidation deficiencies and are speculated to be linked to SCAD-mediated butyric and ethylmalonic acid buildups.

**Fig. 1:**
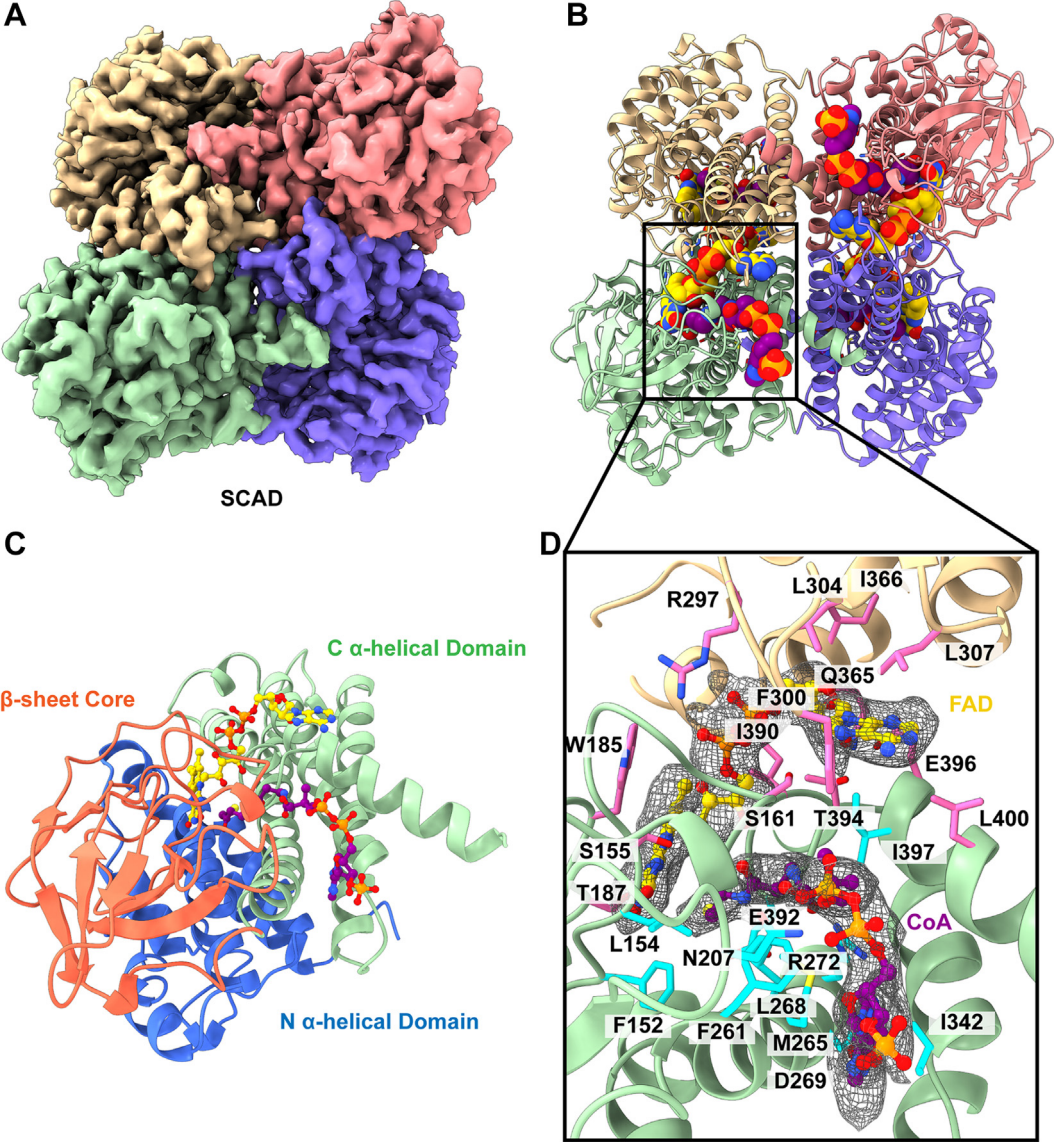
**Cryo-EM structure of human short chain acyl-CoA dehydrogenase.***A*, cryo-EM map of SCAD. The four subunits are colored individually. *B*, ribbon diagram of the structure of SCAD. SCAD forms a D2 symmetric tetramer. Each subunit binds FAD as a cofactor (*yellow sphere* model). A CoA substrate (*purple sphere* model) is also present in the binding cavity of each subunit of SCAD. *C*, structure of a subunit of SCAD. Each subunit contains an N-terminal α-helical domain (*blue*), a middle β-stranded core (*orange*), and a C-terminal α-helical domain (*green*). The FAD- and CoA-binding pockets are formed at the intersection of these three domains. *D*, the FAD- and CoA-binding sites. Cryo-EM densities of bound FAD and CoA are shown in *gray* meshes. Residues responsible for binding FAD are in *pink* sticks, whereas residues responsible for binding CoA are in *cyan* sticks. FAD, flavin adenine dinucleotide; SCAD, short chain acyl-CoA dehydrogenase.

We obtained a high-resolution 3.15 Å structure of SCAD bound to substrate and cofactor using 16,677 single particle cryo-EM projections (Supplemental Table S1, Supplemental Fig. S4, and Fig. 1, *A* and *B*). Our structure is consistent with the previously determined crystal structure of rat SCAD (23) and the unpublished structure of human SCAD (PDB ID: 2VIG). The global fold of SCAD is a tetramer made up of a dimer of dimers. Each individual monomeric unit contains a β-sheet core flanked by N-terminal and C-terminal α-helical domains (Fig. 1*C*). The FAD cofactor-binding site is at the intersection of the three domains of each monomer (Fig. 1*C*). Additionally, the flavin ring interacts with the C-terminal domain of the corresponding subunit in each dimer set. The ligand-binding site lies parallel to cofactor and the catalytic site E392. Residues F152, L154, N207, F261, M265, L268, D269, R272, I342, I397, and the catalytic E392 residue form the substrate cavity (Fig. 1*D*). Interestingly, substrate specificity was altered in a mutagenesis study that altered this catalytic site to correspond to the catalytic site (E286) of IVD (28), another acyl CoA dehydrogenase.

#### Medium chain acyl-CoA dehydrogenase

MCAD has a relatively broad substrate specificity but has highest activity with octanoyl-CoA (29). MCAD deficiency is an autosomal recessive disorder that occurs in roughly 1 out of every 15,000 people (30). Neonatal symptoms can range from hypoglycemia to seizures to sudden infant death if not properly detected (30).

**Fig. 2:**
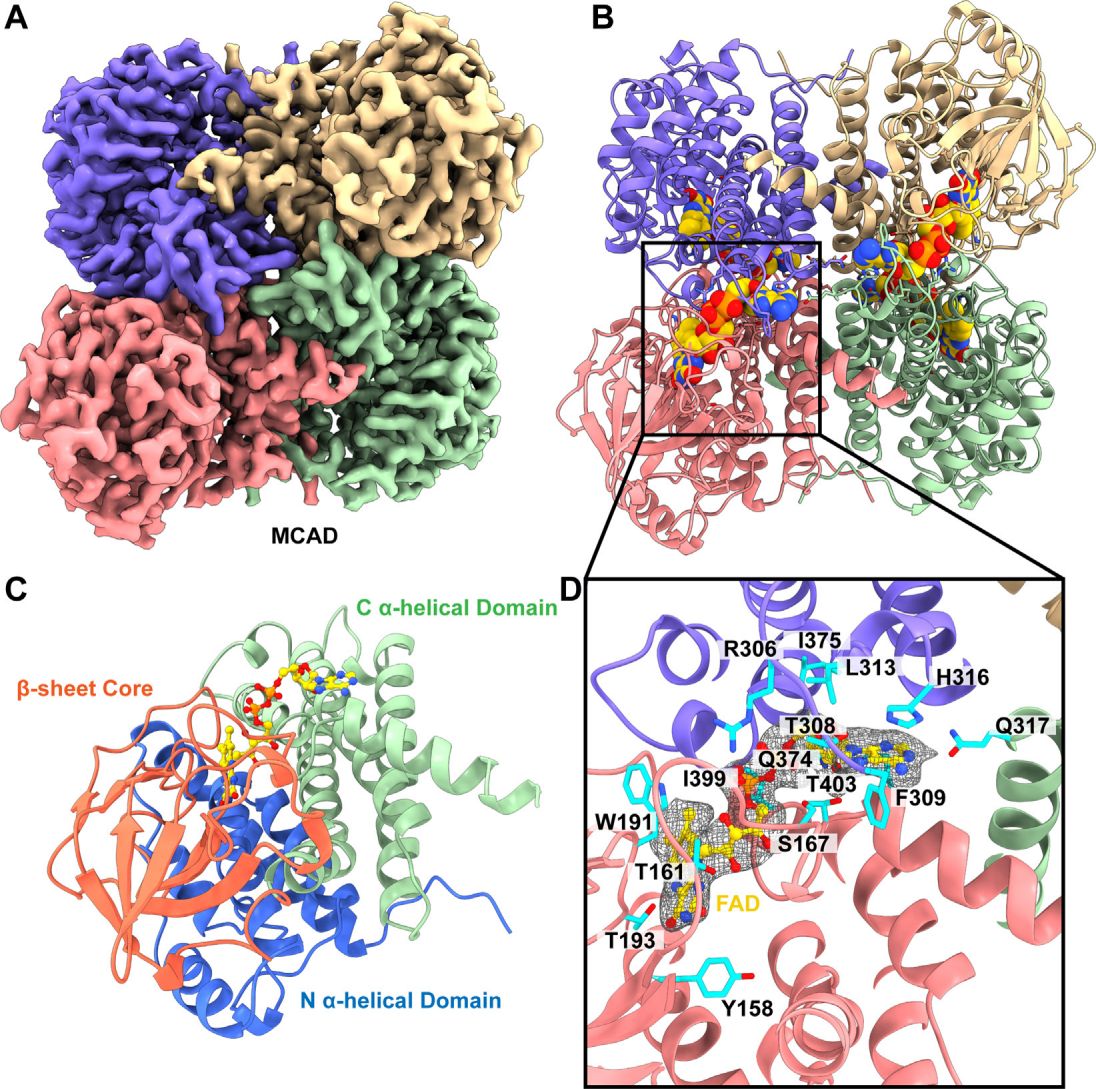
**Cryo-EM structure of human medium chain acyl-CoA dehydrogenase.***A*, cryo-EM map of MCAD. The four subunits are colored individually. *B*, ribbon diagram of the structure of MCAD. MCAD forms a D2 symmetric tetramer. Each subunit binds FAD as a cofactor (displayed as *sphere*). *C*, structure of a subunit of MCAD. Each subunit contains an N-terminal α-helical domain (*blue*), a middle β-stranded core (*orange*), and a C-terminal α-helical domain (*green*). The FAD-binding pocket is formed at the intersection of these three domains. *D*, the FAD-binding site. Cryo-EM density of the FAD cofactor is shown in *gray* mesh. Residues responsible for binding FAD are colored *cyan*. FAD, flavin adenine dinucleotide; MCAD, medium chain acyl-CoA dehydrogenase.

An FAD cofactor-bound MCAD structure was resolved at 2.69 Å using 18,616 single particle cryo-EM projections (Supplemental Table S1, Supplemental Fig. S5, and Fig. 2, *A* and *B*). Consistent with previous crystal structures of human (29) and pig (31) MCAD, our structure is a tetramer consisting of a dimer of dimers. The overall fold is similar to that of SCAD. Each monomer is made up of three domains; an N-terminal α-helical bundle, a β-sheet core, and a C-terminal α-helical domain (Fig. 2*C*). Residues T121, T124, A125, L128, F277, T280, R281, V284, Y398, Y400, and the catalytic residue E401 form the binding cavity (Fig. 2*D*). The substrate accommodation in the binding cavity is determined by a network of hydrogen bonds among residues Q120, T121, Q242, Y398, and Y400. In the MCAD structure, helix 4 and helix 6 are shifted relative to the corresponding SCAD structure. These two helices form the edge of the substrate cavity, resulting in a deeper substrate-binding pocket in the MCAD structure (Supplemental Fig. S6). In turn, this additional depth allows for further flexibility in the accommodation of longer chain fatty acids.

Both the N- and C-terminal helical domains contribute to the helix–helix interactions that form the dimer–dimer interface. Several of the mutations identified in newborn screening of MCAD deficiency (32), including the most common K329E mutation, are not within the active site but instead appear to be involved in the dimer–dimer interface, which would disrupt assembly and limit activity.

#### Isovaleryl-CoA dehydrogenase

IVD is an intermediate enzyme in the leucine catabolism pathway (33). Specifically, IVD catalyzes the conversion of isovaleryl-CoA/3-methylbutanoyl-CoA to 3-methylbut-2 enoyl-CoA. Mutations in the *IVD* gene can result in isovaleric acidemia, a rare autosomal recessive disorder, that results in the buildup of isovaleric acid (34). The disorder typically presents during infancy and can cause severe medical problems such as seizures and coma (35).

**Fig. 3:**
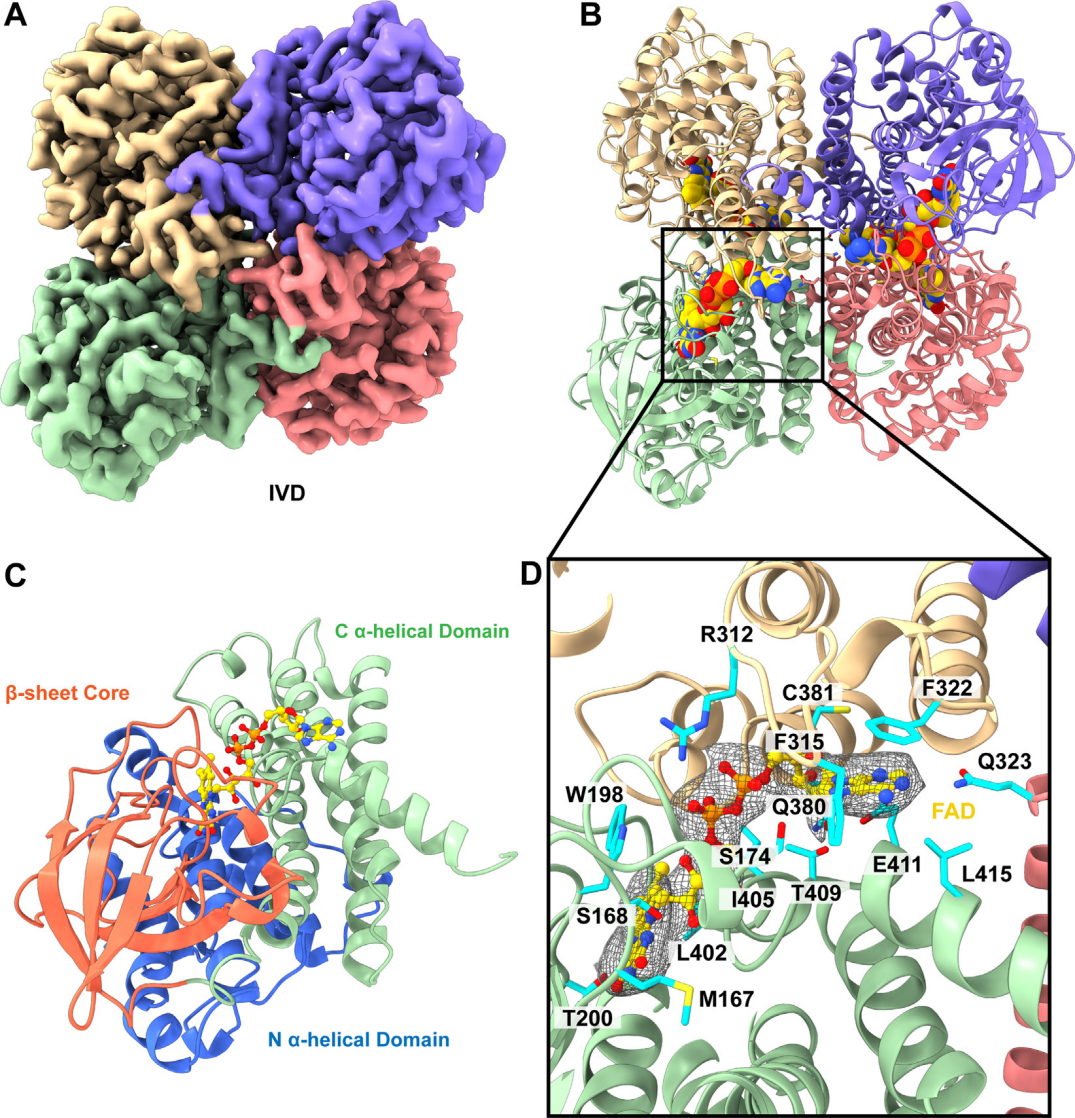
**Cryo-EM structure of human isovaleryl acyl-CoA dehydrogenase.***A*, cryo-EM map of IVD. The four subunits are colored individually. *B*, ribbon diagram of the structure of IVD. IVD forms a D2 symmetric tetramer. Each subunit binds FAD as a cofactor (displayed as *sphere*). *C*, structure of a subunit of IVD. Each subunit contains an N-terminal α-helical domain (*blue*), a middle β-stranded core (*orange*), and a C-terminal α-helical domain (*green*). The FAD-binding pocket is formed at the intersection of these three domains. *D*, the FAD-binding site. Cryo-EM density of the FAD cofactor is shown in *gray* mesh. Residues responsible for binding FAD are colored *cyan*. FAD, flavin adenine dinucleotide; IVD, isovaleryl-CoA dehydrogenase.

A total of 13,185 single particle cryo-EM projections were used to generate a FAD-bound structure of IVD that resolved to 2.84 Å (Supplemental Table S1, Supplemental Fig. S7, and Fig. 3, *A* and *B*). Our structure is consistent with a previous crystal structure (33) and is broadly similar to the structures of SCAD and MCAD with three domains: an N-terminal α-helical domain, a β-sheet core, and a C-terminal α-helical domain (Fig. 3*C*). The FAD cofactor-binding site is located in a position similar to that found in SCAD and MCAD. A key difference among these structures is the binding cavity, which is wider in IVD than in the SCAD and MCAD structures (Fig. 3, *A*–*C*). This is due to the presence of a glycine at position G406 in the IVD molecule as opposed to the corresponding tryptophans Y391 (SCAD) and Y400 (MCAD), which allows for accommodation of the branched substrate. Additional residues that contribute to the binding cavity include L127, A131, L135, T200, L290, L402, Y403, G406, and A407 (Fig. 3*D*). A prior crystal structure also identified E286 as the catalytic base residue, in comparison to E401 in SCAD and MCAD (33).

### Delta (3,5)-Delta (2,4)-Dienoyl-CoA Isomerase (ECH1)

The main beta oxidation pathway uses saturated fatty acids or unsaturated fatty acids with double bonds on even numbered carbons. An auxiliary pathway using ECH1, 2,4-dienoyl-CoA reductase, and delta 3-delta 2-enoyl-CoA isomerase is used to break down unsaturated odd carbon fatty acids (36). The first enzyme in this pathway, ECH1, shifts double bonds from positions 3 and 5 to positions 2 and 4, isomerizing 3,5-dienoyl CoA to 2,4-dienoyl CoA.

**Fig. 4:**
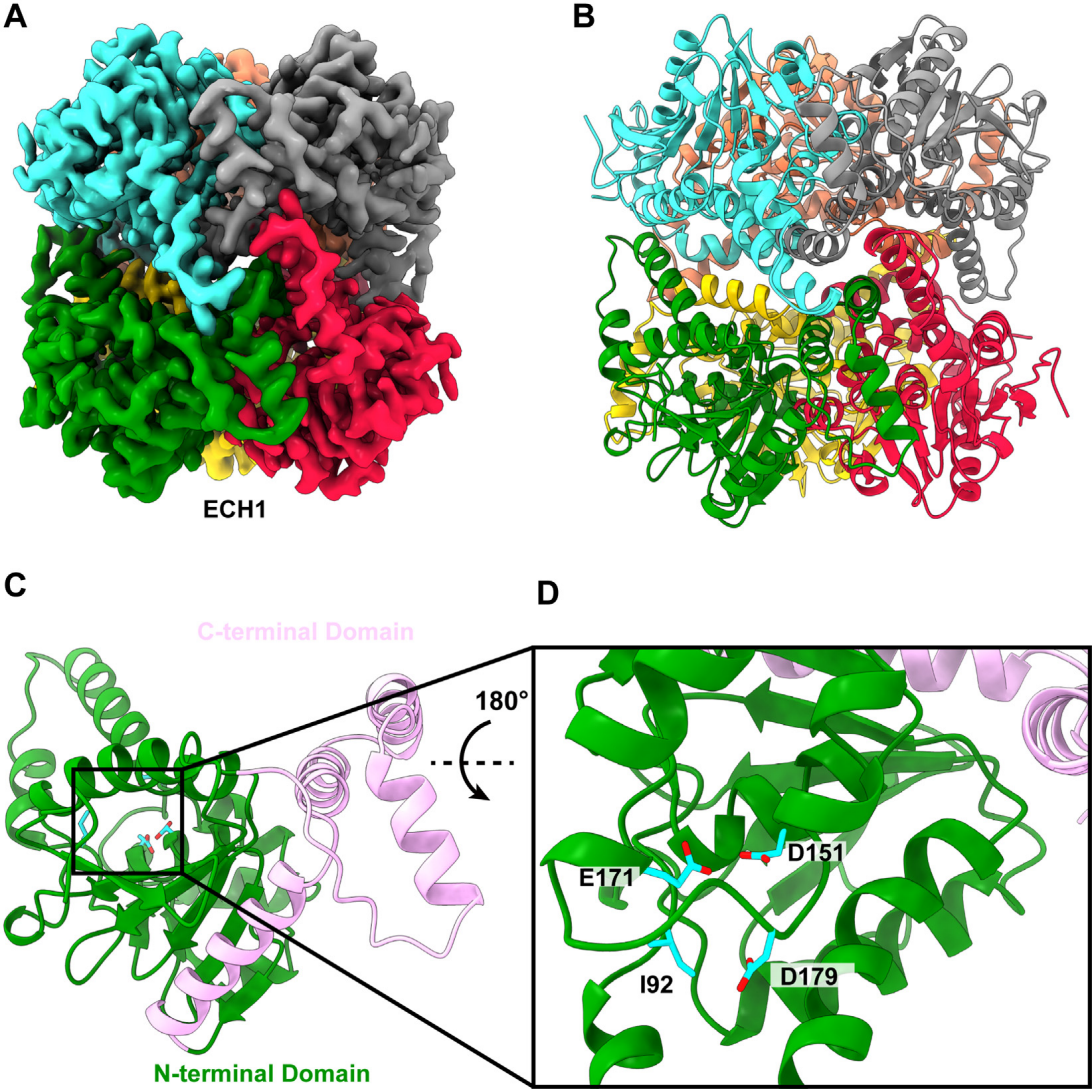
**Cryo-EM structure of human delta(3,5) delta (2,4) dienoyl-CoA isomerase.***A*, cryo-EM map of ECH1. The six subunits are colored individually. *B*, ribbon diagram of the structure of ECH1. ECH1 forms a hexamer made up of two trimers and exhibits D3 symmetry. *C*, structure of a subunit of ECH1. Each subunit contains an N-terminal catalytic domain (*green*) and a shorter C-terminal helical domain (*pink*). *D*, the catalytic-binding site. Residues forming the catalytic site are colored *cyan*.

A total of 8862 single particle cryo-EM projections were collected for this structure. From these projections, we constructed a high-resolution cryo-EM map of 2.96 Å and identified this protein as the ECH1 isomerase (Supplemental Table S1, Supplemental Fig. S8, and Fig. 4, *A* and *B*). Our structure broadly agrees with the existing crystal structure of the rat enzyme (36) and the unpublished crystal structure of the human isomerase (PDB ID: 2VRE). The overall structure is a hexamer made up of a pair of trimers. Each monomer has two domains: an N-terminal catalytic domain and a C-terminal α-helical domain (Fig. 4*C*). Similar to other members of the isomerase family (36), the N-terminal domain is a spiral of four turns. Each turn is made up of two β strands followed by one or two α helices. The C-terminal domain is made up of a core of four α helices that interact with the other two subunits to form the trimerization domain. The trimer–trimer interface is formed by the interaction of the α-helix from the second turn in the catalytic domain interacting with the trimerization domain. We did not observe a bound ligand in our cryo-EM structure but a previously solved rat crystal structure (36) identified equivalent catalytic residues as I118, D177, E197, and D205 (Fig. 4*D*).

### Aspartate Aminotransferase (GOT2)

Humans have two aspartate aminotransferase isoenzymes, the cytosolic GOT1 and the mitochondrial GOT2 enzyme (37). These two enzymes work together with cytosolic and mitochondrial malate dehydrogenases, malate-alpha-ketoglutarate antiporter and glutamate-aspartate antiporter, to form the malate-aspartate shuttle responsible for the movement of the reducing agent, NADH, across the mitochondrial membrane (38). Specifically, GOT2 transfers an amino group from glutamate to oxaloacetate, yielding aspartate and alpha ketoglutarate. Clinically, regulation of GOT2 has been linked to suppression of pancreatic cancer cell growth (39). Additionally, the ratio of aspartate aminotransaminase to alanine aminotransaminase is used as a diagnostic for liver health.

**Fig. 5:**
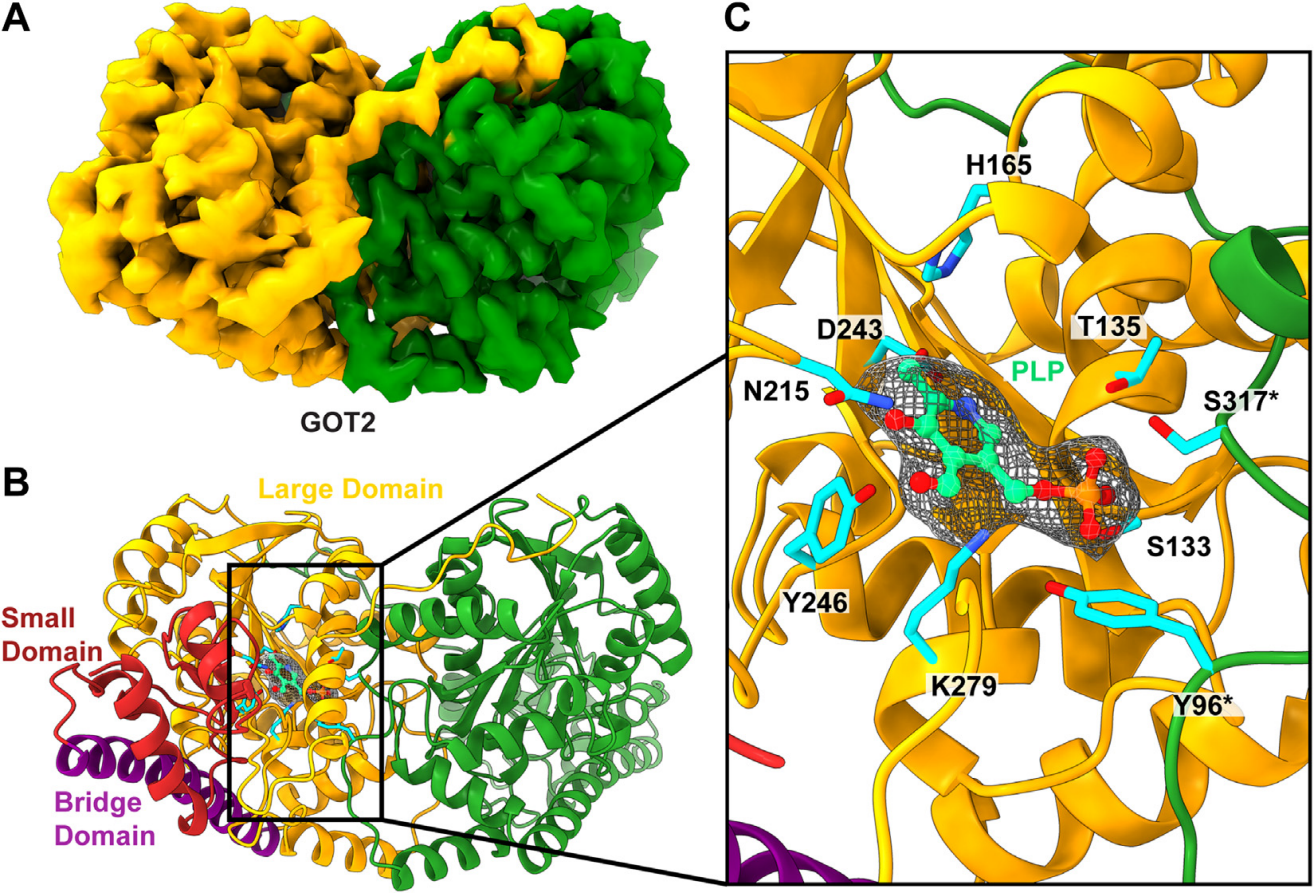
**Cryo-EM structure of human mitochondrial aspartate aminotransferase.***A*, cryo-EM map of GOT2. GOT2 forms a dimer with C2 symmetry. The two subunits are colored individually. *B*, ribbon diagram of the structure of the GOT2 dimer. Each subunit contains a large domain that binds the PLP cofactor (*yellow*), a bridge domain (*purple*), and a small domain (*red*). The second subunit of GOT2 is colored *green*. *C*, the PLP-binding site. This site is formed by residues N215, D243, H165, T135, S133, K279, and Y246 from one subunit (*yellow* cartoon) and Y96′ and S317′ from the other subunit (*green* cartoon). The bound PLP ligand is represented by a ball and stick model. The PLP cryo-EM density is in *gray* mesh. Residues responsible for forming this binding site are colored *cyan*. PLP, pyridoxal phosphate.

We collected a total of 15,723 single particle cryo-EM projections for this protein class. The BaR methodology allowed us to construct a 2.99 Å resolution structure and identify it as aspartate aminotransferase (Supplemental Table S1, Supplemental Fig. S9, and Fig. 5, *A* and *B*). The global structure of GOT2 is a homodimer, consisting of two 45-kDa monomers. Each subunit contains three main domains: a large domain that binds the pyridoxal phosphate (PLP) cofactor, a flexible small domain, and a helical bridge domain (Fig. 5*B*). The large PLP-binding domain consists of a seven stranded beta sheet core bracketed by sets of α helices. The active site is between the large and small domains and is supported by the large domain of the corresponding subunit. Residues S133, T135, H165, N215, D243, Y246, and K279 from one subunit as well as residues Y96′ and S317′ from the other subunit help form the cofactor and active site–binding cavity (Fig. 5*C*). A previous crystal structure also describes a small N-terminal domain (residues 3–14) (37) but our structure did not include this region, likely due to its flexibility.

### Glutamate Dehydrogenase 1

Glutamate dehydrogenase is a mitochondrial matrix protein expressed in high levels in the liver, brain, kidney, heart, pancreas, ovaries, and testes (40). Functionally, mammalian GDH uses NADH or NADPH as a cofactor in order to deaminate glutamate to 2-oxoglutarate, a key intermediate in the Krebs cycle. This catalysis of glutamate also produces ammonium as a secondary product, linking GDH to a role in ammonia detoxification. At the clinical level, mutations in GDH have been linked to a form of familial hyperinsulinism that is characterized by both hypoglycemia and hyperammonemia (41).

**Fig. 6:**
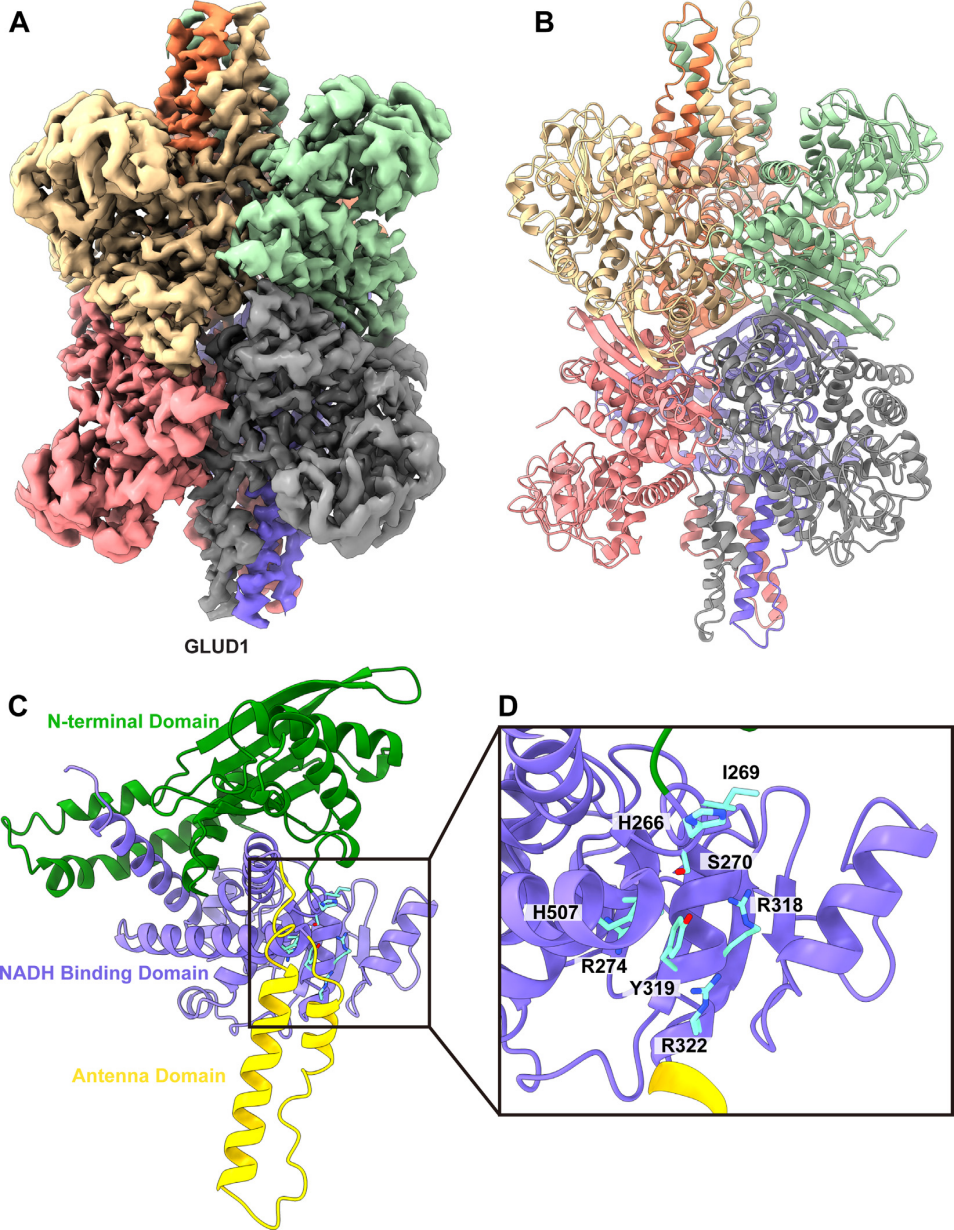
**Cryo-EM structure of human glutamate dehydrogenase 1.***A*, cryo-EM map of GLUD1. The six subunits are colored individually. *B*, ribbon diagram of the structure of GLUD1. GLUD1 forms a hexamer made up of two trimers and exhibits D3 symmetry. *C*, structure of a subunit of GLUD1. Each subunit contains an N-terminal domain (*green*), a NADH-binding domain (*purple*), and a flexible antennae domain (*yellow*). *D*, the GTP-binding site. This binding site is formed by a core of residues (*cyan*) from the NADH-binding domain. GLUD, glutamate dehydrogenase.

We collected a total of 62,508 particles for this class of protein images. From these projections, we were able to construct a 2.31 Å resolution cryo-EM map and identify the structure as the GLUD1 enzyme (Supplemental Table S1, Supplemental Fig. S10, and Fig. 6, *A* and *B*). In agreement with previous human (42) and bovine (43) crystal structures, GLUD1 consists of six subunits that form the functional homohexamer. Each individual subunit consists of an N-terminal glutamate-binding domain (residues 63–266), the NADH-binding domain (267–448 and 528–556), and the antenna domain (449–501) (Fig. 6*C*). The GTP-binding site is bracketed by several key basic residues, including H266, R274, R318, R322, and H507, as well additional residues I269, S270, and Y319 (Fig. 6*D*). There is a large degree of conformational flexibility in both the NADH-binding domain and antenna domain, particularly in the unbound state (42, 43). Additionally, mutations in the antenna domain have also been linked to hyperinsulinism (41, 42).

### Mitochondrial SOD2

Superoxide, O_2_^−^, is produced following the reduction of oxygen during aerobic respiration and is the primary reactive oxygen species in the mitochondrial matrix (44). High concentrations of superoxide and other reactive oxygen species are toxic and contribute to cellular apoptosis (45). SOD enzymes are broadly classified by their associated metal cofactors. SOD2 is a manganese-binding member of the SOD family that is expressed in the mitochondria and catalyzes the oxidation of two O_2_^−^ ions to molecular O_2_ and H_2_O_2_ (46). Removal of O_2_^−^ is critical in limiting damage due to cell death–inducing oxidative stresses during cardiac myopathies such as ischemic heart disease (45). Additionally, regulation of SOD2 has been implicated in cancer, metabolic and neurodegenerative diseases, and aging (44, 45).

**Fig. 7:**
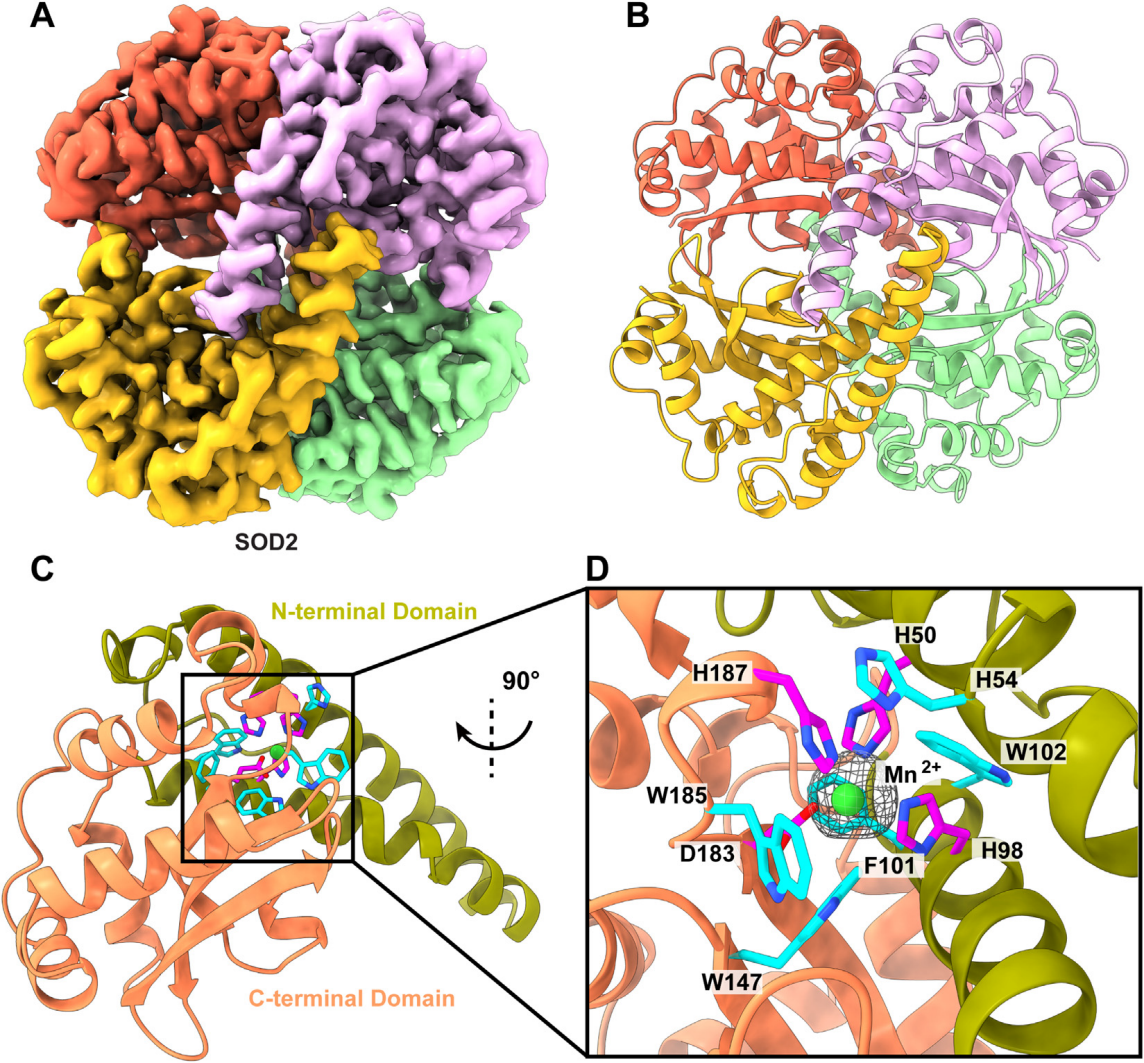
**Cryo-EM structure of human mitochondrial superoxide dismutase 2.***A*, cryo-EM map of SOD2. The four subunits are colored individually. *B*, ribbon diagram of the structure of SOD2. SOD2 forms a D2 symmetric tetramer. *C*, structure of a subunit of SCAD. Each subunit contains an N-terminal domain (*olive green*) and a C-terminal domain (*salmon*). *D*, the Mn^2+^-binding site. Each subunit of SOD2 binds one Mn^2+^ ion. The bound Mn^2+^ ion is coordinated by three histidine residues (H50, H98, and H187) and one aspartate residue (D183). These residues are colored *magenta*. An additional stabilizing hydrophobic pocket is formed by residues H54, F101, W102, W147, and W185. SCAD, short chain acyl-CoA dehydrogenase; SOD, superoxide dismutase.

Our BaR methodology classified a total of 50,014 single particle projections for the SOD2 class. Based on these projections, we constructed a high-resolution density map of 2.91 Å (Supplemental Table S1, Supplemental Fig. S11, and Fig. 7, *A* and *B*). The global architecture of SOD2 is a homotetramer formed by a dimer of dimers. Similarly to existing crystal structures (47, 48), each subunit of SOD2 contains an N-terminal domain primarily consisting of two long α helices and a C-terminal domain made up of a core three stranded β sheet flanked by a set of six α helices (Fig. 7*C*). A central cavity is formed by the interface of each subunit’s β-sheet core, while dimer interfaces consist of α-helical bundles from the N-terminal domains. Elements of both domains contribute to the Mn^2+^-binding site for each subunit. Specifically, this cavity is formed by the H50 and H98 from the N-terminal domain and D183 and H187 from the C-terminal domain. An additional stabilizing hydrophobic pocket is formed by residues H54, F101, W102, W147, and W185 (Fig. 7*D*).

### Catalase

Hydrogen peroxide, H_2_O_2_, is a toxic byproduct of many metabolic pathways. CAT is an enzyme found in many organs whose main function is to break down hydrogen peroxide into water and oxygen to prevent H_2_O_2_-induced damage (49). Given this ubiquitous role, CAT activity has been linked to inflammation, cell death, aging, and cancer (50). Acatalasemia, an autosomal recessive disorder, characterized by oral ulcers, is also caused by significantly reduced levels of CAT (51).

**Fig. 8:**
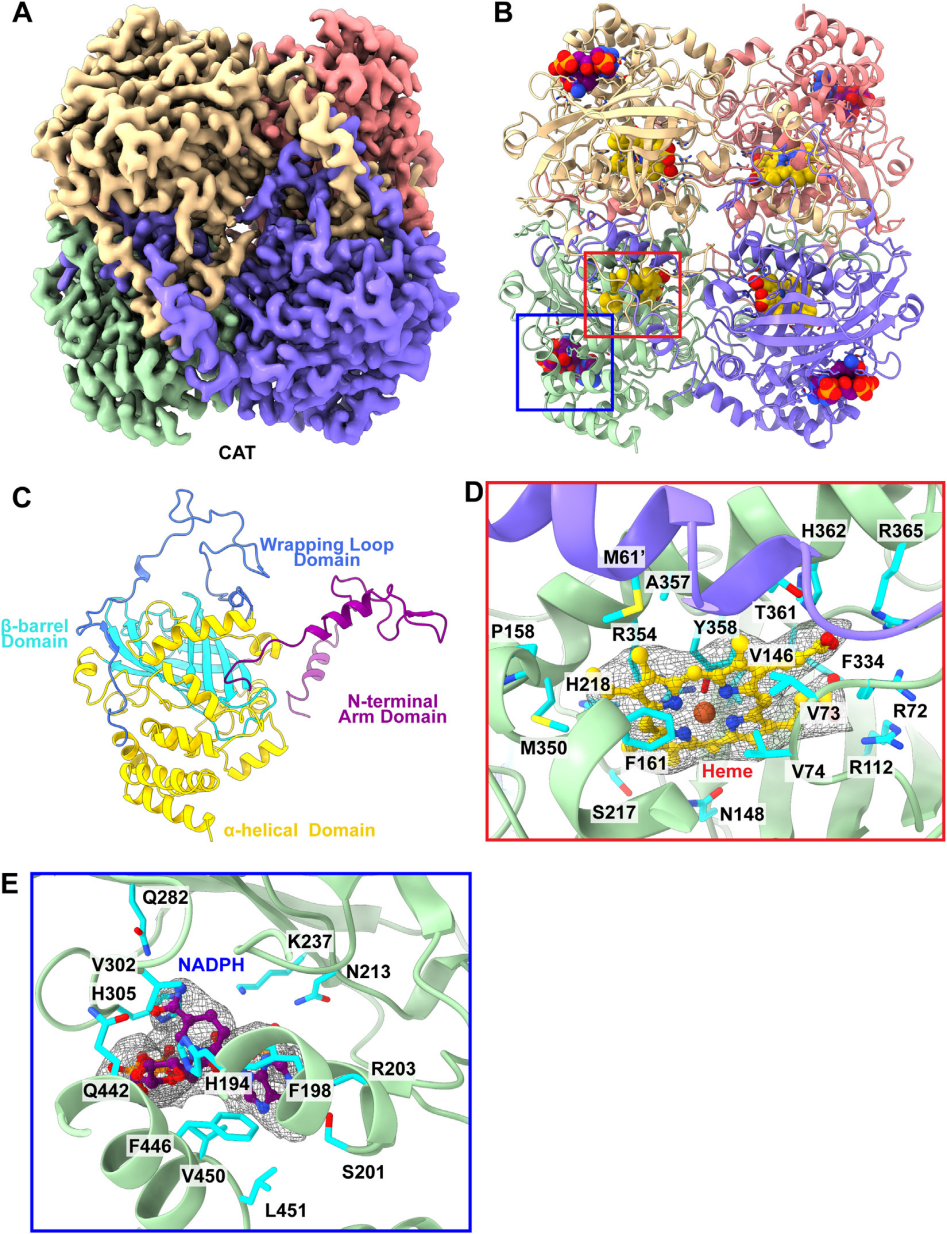
**Cryo-EM structure of human catalase.***A*, cryo-EM map of CAT. Each subunit of the CAT tetramer is colored individually. *B*, ribbon diagram of the structure of CAT which forms a D2 symmetric tetramer. Each subunit binds NADPH as a cofactor (*purple sphere* model). Each subunit also binds one heme (*yellow sphere* model). *C*, structure of a subunit of CAT. Each subunit consists of an N-terminal arm domain (*purple*), a β-barrel domain (*cyan*), a wrapping loop domain (*blue*), and a C-terminal α-helical domain (*yellow*). *D*, the heme-binding site. This heme active site is bordered by residues R72, V73, V74, R112, V146, N148, P158, F161, S217, H218, F334, M350, R354, A357, A358, T361, H362, and R365 from one subunit (*green* cartoon). A second subunit (slate cartoon) extends an N-terminal helix with residue M61′ to cover the active site. The cryo-EM density of bound heme is in *gray* mesh. *E*, the NADPH-binding site. The bound NADPH molecule is colored *purple*. Its cryo-EM density is in *gray* mesh. Residues involved in binding NADPH are colored *cyan*. CAT, catalase.

We collected a total of 31,971 single-particle cryo-EM projections for this class of protein images. The BaR methodology allowed us to construct a high-resolution cryo-EM map. Subsequently, we were able to identify this protein as the CAT enzyme and resolve its structure to a resolution of 2.58 Å (Supplemental Table S1, Supplemental Fig. S12, and Fig. 8, *A* and *B*). Consistent with existing crystal structures of liver CAT (49), each CAT subunit contains four distinct domains: a β-barrel domain, an α-helical domain, a N-terminal arm domain, and a wrapping loop domain (Fig. 8*C*). The hydrophobic core of the subunit is made up of a β-barrel domain made of eight antiparallel β sheets. Eight α helices, including four C-terminus helices and four located between β sheets β4 and β5, form the α-helical domain of each subunit. Two subunits are linked by threading the N-terminal arm of each subunit through the wrapping loop domain of the other subunit. Two sets of linked subunits assemble to form the tetramer in a manner where the N-terminal arms cover the heme active sites. A long channel in the β barrel domain leads to each of the heme active site, which consists of the residues M61, R72, V73, V74, R112, V146, N148, P158, F161, S217, H218, F334, M350, R354, A357, Y358, T361, H362, and R365 (Fig. 8*D*). The NADPH-binding site rests between the α-helical and β-barrel domains and is stabilized by residues H194, F198, S201, R203, N213, K237, Q282, V302, H305, Q442, F446, V450, and L451 (Fig. 8*E*).

### Mitochondrial ALDH2

Mitochondrial ALDH is localized in the mitochondrial matrix with a primary role in metabolizing acetaldehyde to acetate in the alcohol metabolism pathway (52). Alcohol flushing syndrome, which is characterized by dizziness, nausea, facial flushing, and tachycardia following ethanol consumption, is the result of a dominant negative single point mutation (E487K) (53). This mutation results in a stable, but significantly less active, protein product leading to accumulation of aldehydes in the bloodstream (53, 54). More specifically, this single point mutation results in an increased K_m_ for the cofactor NADH. Recently, this ALDH2 mutation has been reported as a risk factor for late-onset Alzheimer’s disease, potentially *via* a buildup of reactive oxidative species (52, 55).

**Fig 9:**
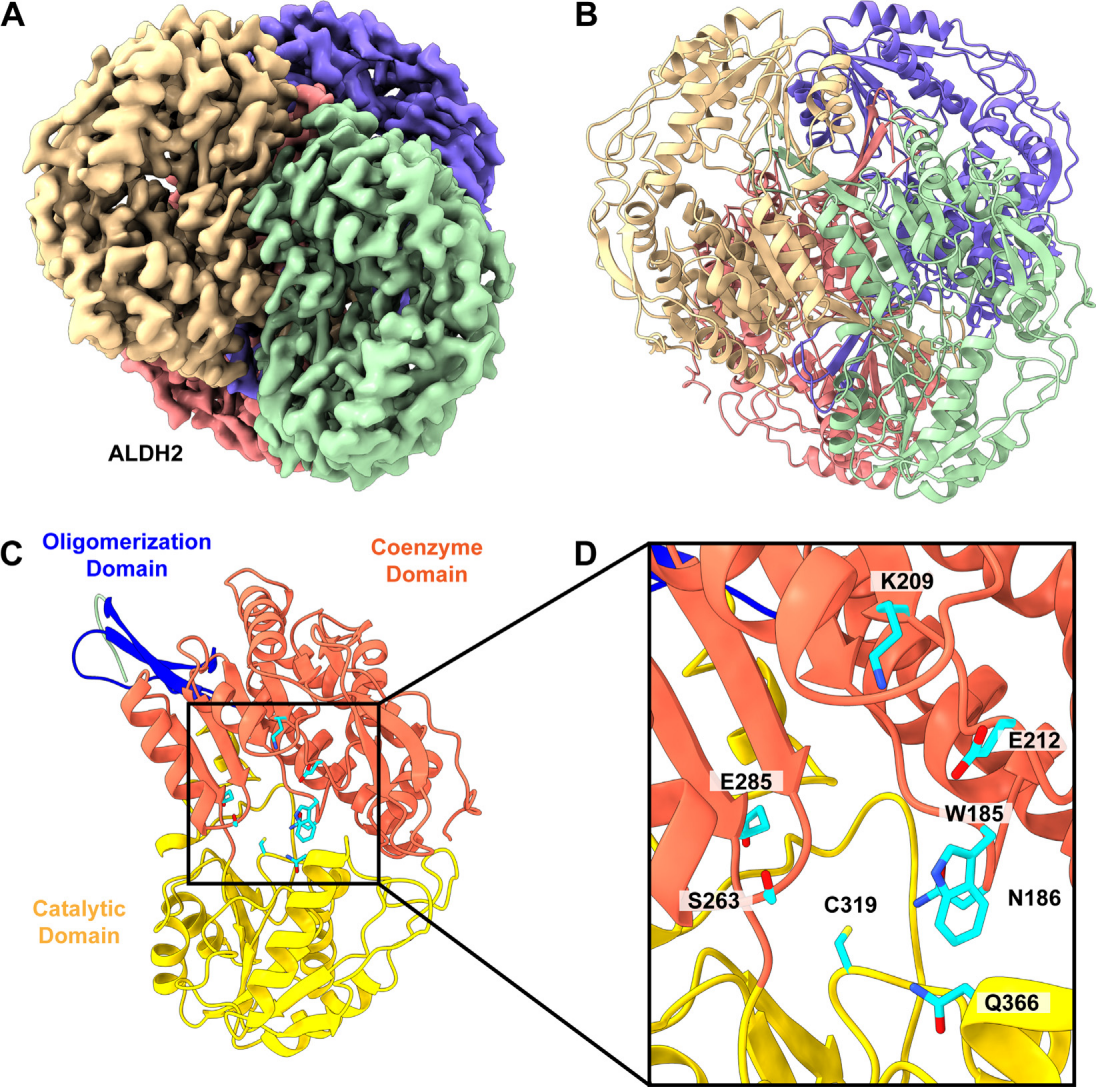
**Cryo-EM structure of human mitochondrial aldehyde dehydrogenase 2.***A*, cryo-EM map of ALDH2. Each subunit is colored individually. *B*, ribbon diagram of the structure of GOT2. GOT2 forms a tetramer with D2 symmetry. *C*, structure of a subunit of ALDH2. Each subunit contains a catalytic domain (*yellow*), a coenzyme-binding domain (*orange*) and an oligomerization domain (*blue*). *D*, the catalytic site of ALDH2. Residues forming the catalytic site are in *cyan* sticks. ALDH, aldehyde dehydrogenase.

We collected a total of 82,360 single-particle cryo-EM projections for this class of protein images. Based on these projections, the BaR methodology allowed us to construct a high-resolution cryo-EM map. Subsequently, we were able to identify this protein as the ALDH2 enzyme and resolve its structure to a resolution of 2.66 Å (Supplemental Table S1, Supplemental Fig. S13, and Fig. 9, *A* and *B*). The structure of ALDH2 is a dimer of dimers, forming a functional tetramer.

Matching with existing crystal structures (54) of the ALDH2 enzyme, each ALDH2 subunit consists of a coenzyme domain, a catalytic domain, and an oligomerization domain (Fig. 9*C*). The coenzyme domain (24–155 and 176–287) possesses an NADH-binding site. Both the cofactor and catalytic domain (residues 288–502) contain a five stranded α/β sheet followed by two antiparallel β strands. The oligomerization domain (156–175 and 503–511) is a three stranded antiparallel β sheet. Presumably, residues W185, N186, K209, E212, S263, Q366, E285, and C319 are engaged in NADH binding and C319 is the catalytic residue (Fig. 9*D*). However, we did not observe bound NADH in our cryo-EM structure.

## Discussion

Cellular processes are made up of a complex concert between protein, small molecule, and enzyme interactions. A comprehensive systems biology approach is highly desirable at the structural level, as it offers a holistic understanding of the underlying biological interactions. We previously developed a BaR methodology (9) in order to elucidate multiple protein components of intricate cellular systems from a single biological sample. In our present study, we used BaR to simultaneously identify and solve high-resolution cryo-EM structures of nine important mitochondrial enzymes to resolutions between 2.31 Å and 3.15 Å (Supplemental Table S1). The identity of these enzymes was confirmed by proteomics using LC-MS/MS (Supplemental Table S2).

As with previous BaR applications (9, 56, 57), our methodology was able to identify and solve structures of proteins with relatively low (<10%) sample populations. By adding an additional 3D classification to our BaR pipeline, we also were able to distinguish and solve high-resolution structures of three very similar enzymes with 32 to 38% amino acid sequence similarity from the acyl-CoA dehydrogenase enzyme family. This result highlights the capability of BaR to discriminate between very similar proteins of the same class and extracting their unique structural information.

Although all of these nine solved enzyme structures are symmetrical in nature and each with identical protein subunits, the BaR methodology is also able to identify and determine structures of asymmetric proteins and complexes. This is evident from previous work where BaR was employed to simultaneously solve cryo-EM structures of the asymmetric multiprotein complex cytochrome bo_3_ of *Escherichia coli* and the monomeric HpnN transporter of *Burkholderia pseudomallei* (9). In addition, BaR can simultaneously reveal different conformational states of a particular protein in a single grid. This has been shown clearly in the studies of the *Acinetobacter baumannii* AdeB and *E. coli* CusA transporters (58, 59).

BaR can also be used to identify and characterize endogenous cofactor, ligand, and/or ion binding. For example, in the present study, cofactors and substrates of several mitochondrial enzymes were detected. These include FAD cofactor binding in our trio of identified acyl-CoA dehydrogenases, NADPH cofactor binding to CAT, PLP binding to GOT2, and butanoyl-CoA substrate binding to SCAD.

Posttranslational oligosaccharide modifications play a critical role in localization, solubility, stability, and function of many proteins and enzymes. Besides the identification of bound ligands and ions, BaR can enable us to elucidate structures of posttranslational modifications, glycosylations, and cysteine modifications of enzymes, based on the cryo-EM maps, and these modifications can be validated by mass spectrometry. The ability of BaR to disclose posttranslational modification sites of enzymes has been clearly demonstrated in the work of human liver enzymes (60), where the human hexose-6-phosphate dehydrogenase, glucosidase II, and carboxylesterase 1 were found to be glycosylated.

It is exciting and worth mentioning that our nine enzymes operate at different junctions of the energy production pathway and BaR can help catch a glimpse of these enzymes concurrently. Breakdown of fatty acids begins *via* a dehydration step (61). SCAD and MCAD perform this initial step for short and medium fatty acid chains, respectively. While saturated and unsaturated fatty acids with double bonds at even numbered carbons enter the main beta-oxidation pathway, unsaturated fatty acids with double bonds at odd numbered carbons must first be modified (62). ECH1 catalyzes the first step in this auxiliary pathway. Amino acid catabolism occurs in parallel to the fatty acid metabolism pathway. A third acyl-CoA dehydrogenase enzyme, IVD, is one enzyme in a pathway that breaks down leucine into acetyl-CoA and acetoacetate (20, 33).

Following fatty acid, protein and carbohydrate breakdown acetyl CoA is used in the citric acid cycle to generate ATP. This cycle reduces NAD^+^ to NADH. Recycling of NADH is mediated by GOT2, which also plays a role in the interconversion of aspartate and α-ketoglutarate to oxaloacetate and glutamate (63). Respiration generates toxic byproducts such as reactive oxidative species and hydrogen peroxide (44). SOD2, CAT, and ALDH2 play stepwise roles in helping regulate the buildup of these toxic metabolites. First, SOD2 transforms O_2_^−^ into H_2_O_2_ and O_2_. Then, CAT uses H_2_O_2_ to catalyze the oxidation of several toxins including acetaldehyde, which can be further converted to acetic acid by ALDH2 (50, 54).

Even though we did not observe any previously undefined enzyme structures in this study and the structures of the nine identified enzymes have been characterized before, BaR is capable of identifying and solving structures of novel proteins even if the populations of corresponding particles are as low as 5%. This methodology is also able to determine previously unidentified enzyme complexes, allowing us to understand how different enzymes interact within the native tissue sample. Indeed, the BaR methodology has been used to solve the first structure of the human endoplasmic bifunctional enzyme hexose-6-phosphate dehydrogenase (60). It has also been utilized to obtain the first structures of the human heterodimeric enzyme glucosidase II complex (60) and the human peroxiredoxin 4—endoplasmic reticulum protein 46 complex (60). Therefore, future experiments with modified mitochondrial sample preparations and enrichment protocols will likely reveal additional protein structures/complexes not found in this study.

Although the field of using cryo-EM to elucidate systems proteomics and interactomes is still under development, there are a few cryo-EM methodologies currently available for studying the structural biology of cell lysates. Among them, BaR is unique in that it can offer a possibility to simultaneously solve high-resolution structures of a number of relatively small (<100 kDa) and less abundant (<10%) unidentified proteins within a heterogeneous protein mixture sample. Nonetheless, our application of BaR on soluble mitochondrial organelles is only a small slice of the potential systems biology applications and there are several limitations in this methodology. First, proteins with cryo-EM maps of >4.0 Å remain difficult to identify. Second, it is difficult, if not impossible, to identify a particular protein if the particle population of the protein is <5% in the heterogeneous sample. In addition, preferential orientation of protein images can be a significant problem, as BaR may not be capable of finding a solution for a particle class with insufficient orientation views. Indeed, in our study, there were several unique structures that we were unable to identify. This is most likely the result of preferential orientation, which limits the ability to build an initial starting model and affects the downstream resolution.

It is interesting to note that we did not observe a strong correlation between the population ranking of proteins *via* mass spectrometry and cryo-EM single particle counts. This is surprising as both BaR and LC-MS/MS are based on analyzing and counting protein populations. However, this discrepancy could be easily explained. It could be attributed to additional steps for the preparation of cryo-EM grids for single-particle imaging. As an example, the procedures for preparing a cryo-EM grid include a blotting process, where the filter paper may have different absorptivity for different proteins. During blotting, a large population of proteins may be absorbed by the filter paper, thus significantly altering the composition of protein components and their relative populations on the grid.

In this study, we only describe structures from healthy tissue. Given the prominent role of our subset of identified enzymes in metabolic disorders, this offers an exciting potential of using BaR to compare healthy and mitochondria from different disease phenotypes. This approach could help uncover salient information regarding the mechanism of a particular disease. In addition, our mitochondria were derived from the liver. Comparing mitochondria from different tissue types can possibly be another research approach that will complement existing proteomics methodologies.

Additionally, we believe that BaR can help facilitate the investigation of membrane proteome of different cell membranes. Membrane proteins constitute approximately 30% of the human proteome, and they are often found to be important targets for drug discovery. The ability of using BaR to simultaneously identify and solve high-resolution structures various membrane proteins has been clearly demonstrated in the experimental study of *E. coli* K12 cell membrane (9). After going through the procedures of BaR, the top two most abundant membrane protein particles within the membrane sample were identified to be the outer membrane osmoporin channel OmpC and succinate-coenzyme Q reductase SQR membrane protein complex. The cryo-EM structures of this protein and protein complex were solved to high resolutions of 2.56 Å and 2.50 Å, respectively (9).

Our BaR platform is designed for using cryo-EM to study tissue and organ samples in the context of elucidating systems proteomics and protein–protein interaction partners. However, we still have a mountain to climb to achieve the goal of simultaneous identification and determination of structures of hundreds of proteins in a single sample. Nevertheless, our work strongly indicates that we can utilize BaR and cryo-EM to solve structures of a variety of enzymes from a human organ sample at high resolutions. We believe that BaR can complement affinity purification coupled with mass spectrometry (64) and proximity-dependent biotinylation identification (65) to decipher protein-protein interaction networks and mapping networks of signal transduction to atomic resolutions. Coupled with different techniques in proteomics and interactomics, including those based on cross linking, affinity purification, and cofractionation, it is expected that cryo-EM will enable a new era and perspective in the elucidation of the human proteome and interactome at the atomic level.

## Data availability

Coordinates and EM maps for SCAD, MCAD, IVD, ECH1, GOT2, GLUD1, SOD2, CAT, and ALDH2 can be found at PDB accession numbers 8SGS, 8SGP, 8SGR, 8SK6, 8SKR, 8SK8, 8SKS, 8SGV, and 8SHS and EMDB accession codes EMD-40466, EMD-40463, EMD-40465, EMD-40556, EMD-40565, EMD-40558, EMD-40566, EMD-40469, and EMD-40493, respectively. The MS proteomics data have been deposited to the ProteomeXchange Consortium (http://proteomecentral.proteomexchange.org) *via* the PRIDE partner repository (66) with the data set identifier PXD044860.

## Supplemental data

This article contains supplemental data.

## Conflict of interest

All authors declare that they have no competing interests.
