# Supplementary material for: High-Resolution Structural Proteomics of Mitochondria Using the ‘Build and Retrieve’ Methodology
Source: Mol Cell Proteomics. 2023 Oct 14;22(12):100666. doi: 10.1016/j.mcpro.2023.100666 (PMC10709515; doi:10.1016/j.mcpro.2023.100666)
Supplement: Supplemental Figures S1–S13 and Tables S1 and S2 [file mmc1.pdf]

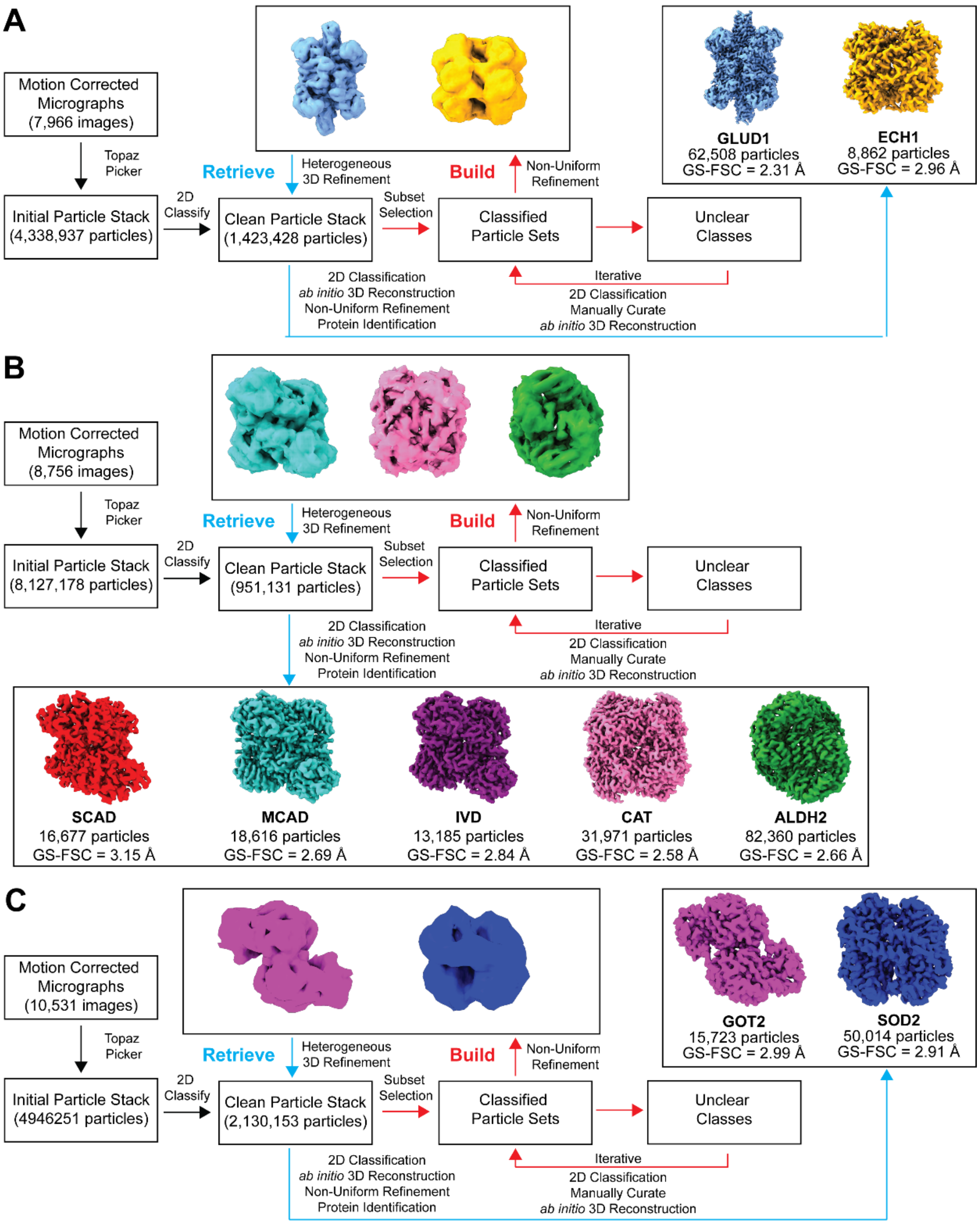

Figure S1. Build-and-Retrieve workflow. (A) Workflow for mitochondrial proteins in 200-450 kDa range. Cryo-EM processing begins as standard workflow, as motion-corrected micrographs are picked, particles undergo 2D classification and initial models are iteratively built. These low-resolution initial models are then used to retrieve particles from the cleaned dataset, resulting in 2 high-resolution maps from the 200-450 kDa mitochondrial proteins: GLUD1 and ECH1. (B) Similar workflow for the 150-250 kDa mitochondrial proteins resulted in 5 high-resolution maps: SCAD, MCAD, IVD, CAT and ALDH2. (C) Similar workflow for the 80-50 kDa mitochondrial proteins resulted in 2 high-resolution maps: GOT2 and SOD2.

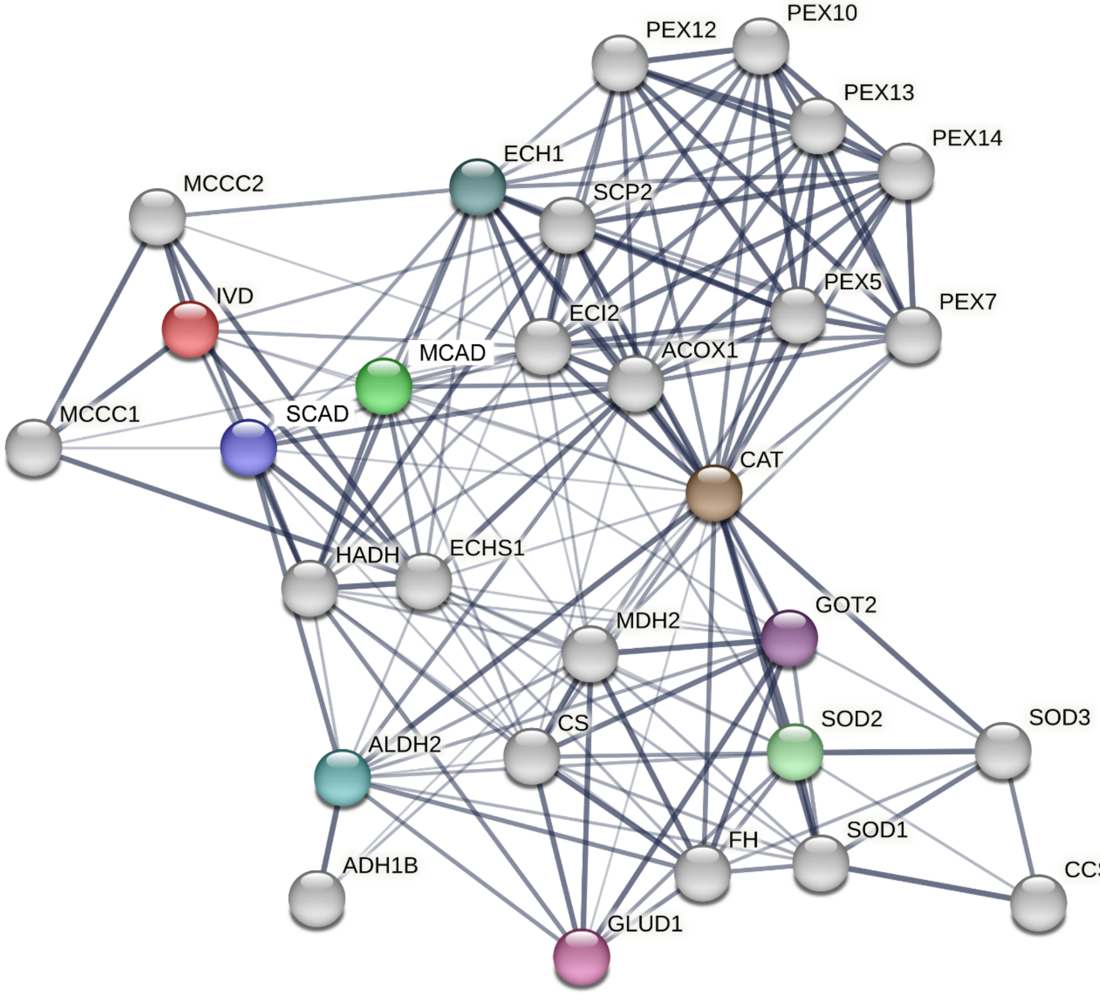

Figure S2. Protein interaction network of mitochondria. This interaction network is created using the STRING database. Line thickness depicts interaction confidence and view was expanded to show 100 interactions. Results show all proteins identified from BaR interact through a complex network of proteins. The nine proteins, SCAD, MCAD, IVD, ECH1, GOT2, GLUD1, SOD2, CAT and ALDH2, are highlighted by colored nodes.

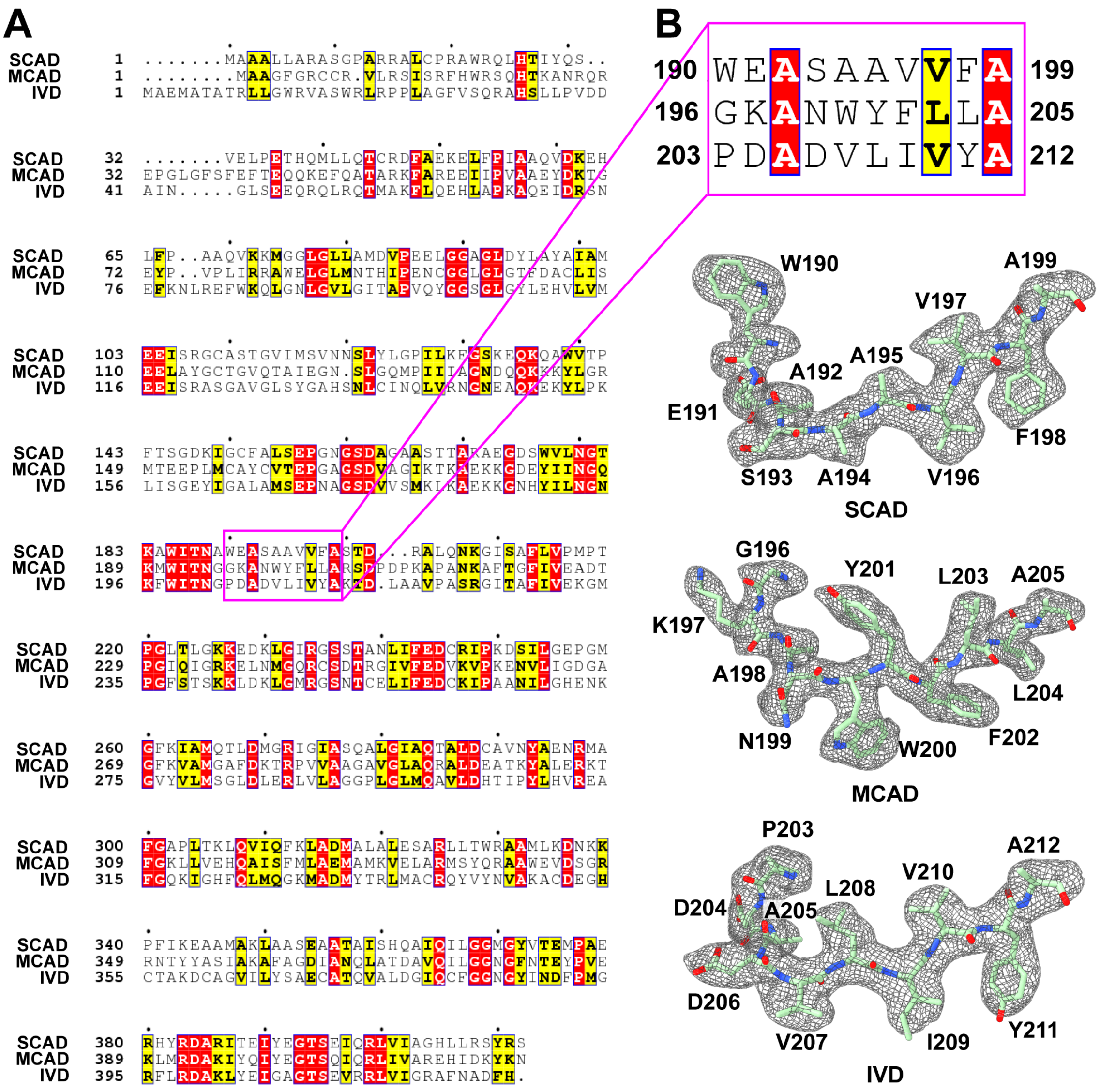

Figure S3. The SCAD, MCAD and IVD acyl-coenzyme A dehydrogenases. (A) Protein sequence alignment of SCAD, MCAD and IVD. Pairwise alignments reveal protein sequence identity between 32% and 38% for these three proteins. (B) Local cryo-EM density maps of SCAD, MCAD and IVD. The 10 corresponding amino acids of these three acyl-coenzyme A dehydrogenases are listed above the maps. The cryo-EM maps are indicated as gray meshes. The corresponding amino acids are in green sticks.

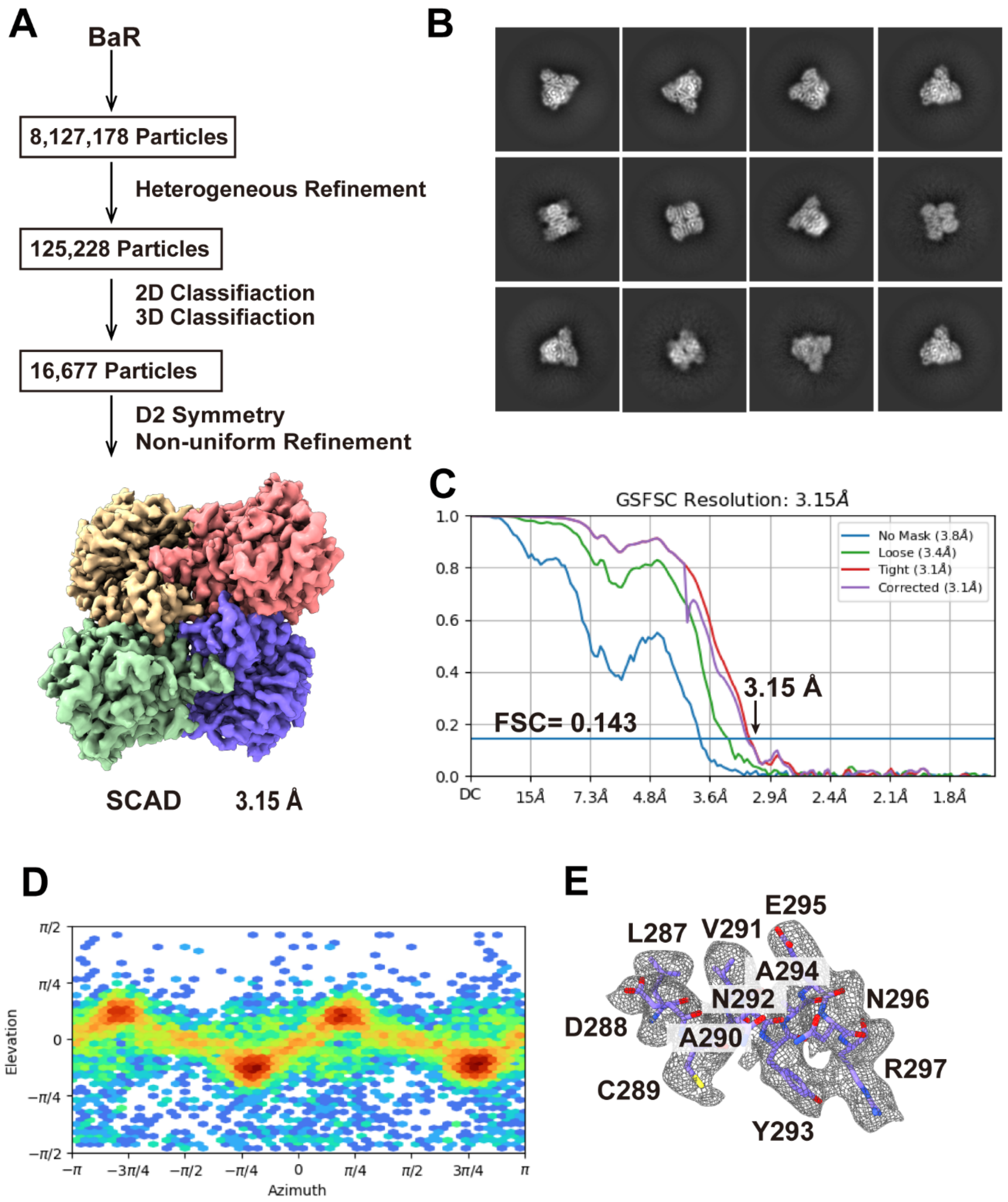

Figure S4. Cryo-EM structural determination of SCAD. (A) Particle stack workflow. (B) Representative 2D classes. (C) Fourier shell correlation curves, cutoff 0.143. (D) Angular distribution calculated in cryoSPARC. (E) Local representative cryo-EM density map.

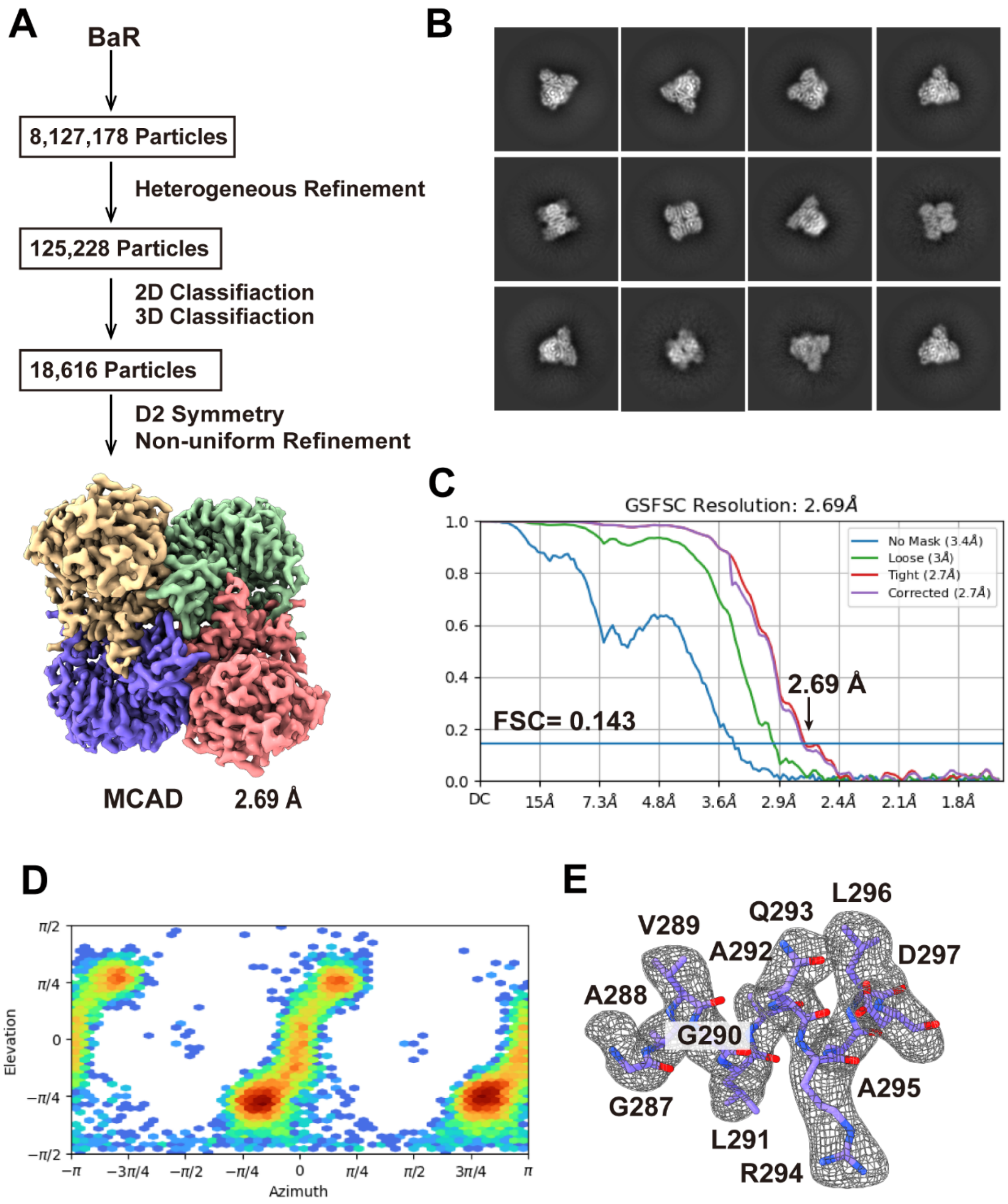

Figure S5. Cryo-EM structural determination of MCAD. (A) Particle stack workflow. (B) Representative 2D classes. (C) Fourier shell correlation curves, cutoff 0.143. (D) Angular distribution calculated in cryoSPARC. (E) Local representative cryo-EM density map.

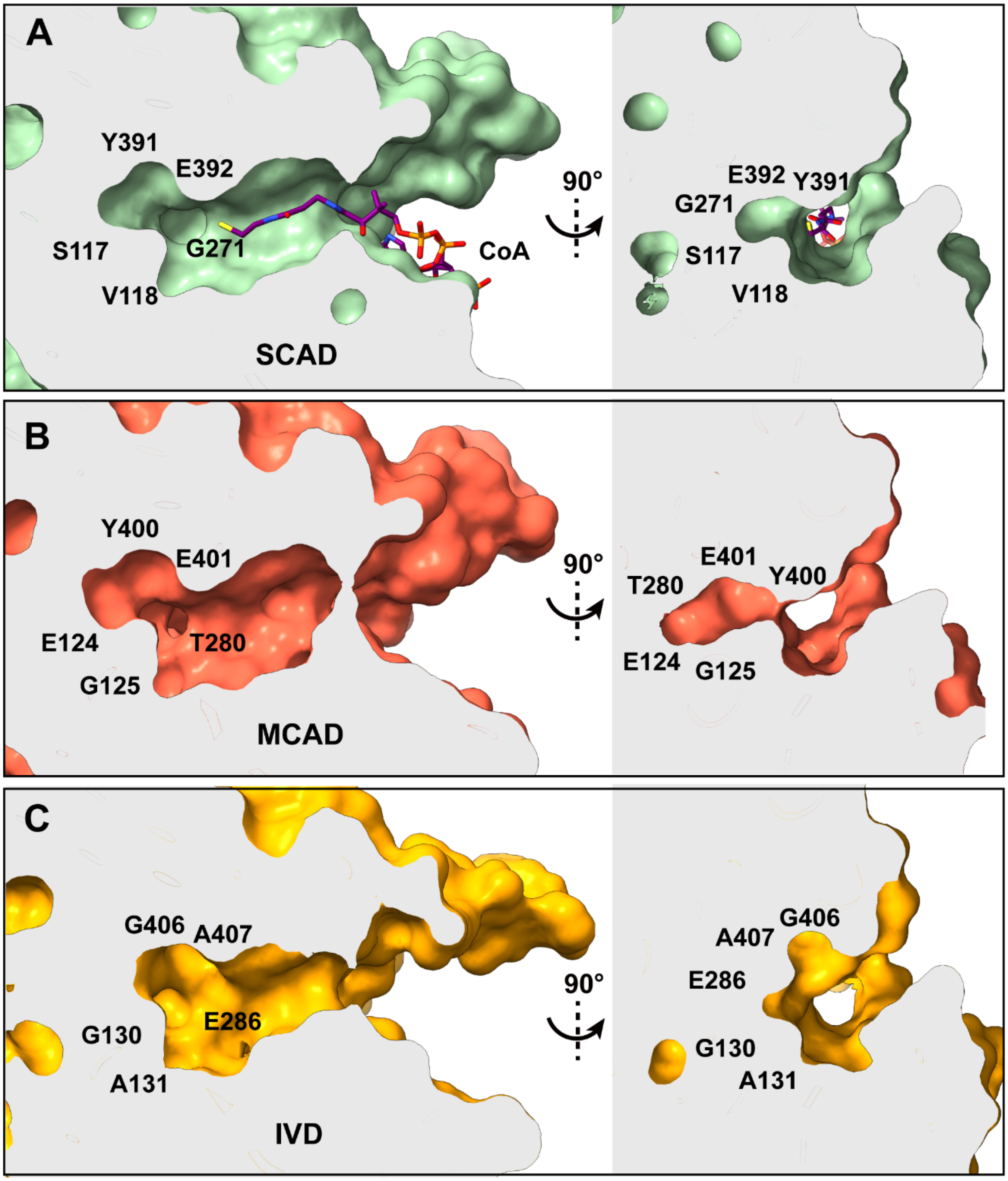

Figure S6. Substrate binding pocket comparisons among SCAD, MCAD and IVD. (A) Substrate binding pocket of SCAD. (B) Substrate binding pocket of MCAD. (C) Substrate binding pocket of IVD.

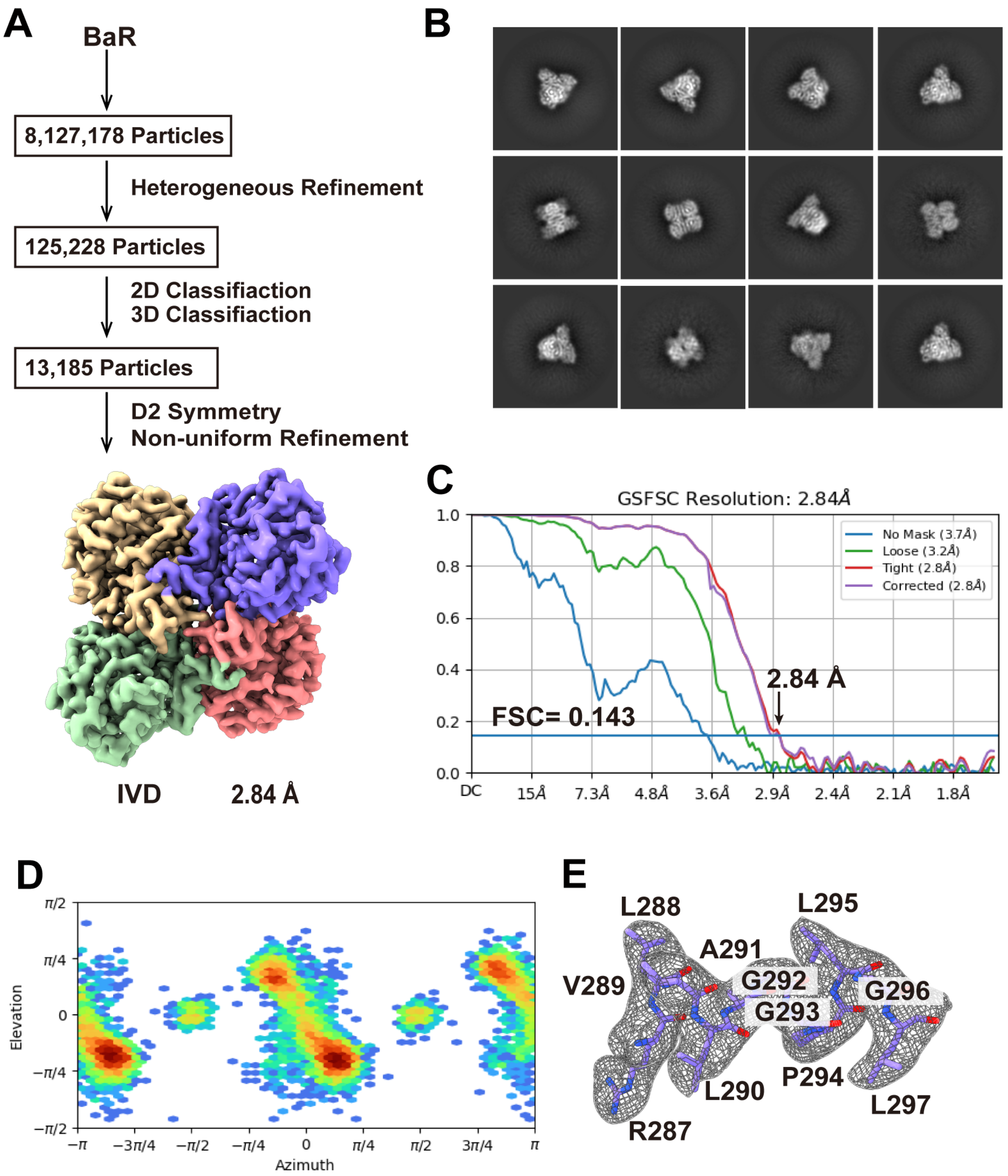

Figure S7. Cryo-EM structural determination of IVD. (A) Particle stack workflow. (B) Representative 2D classes. (C) Fourier shell correlation curves, cutoff 0.143. (D) Angular distribution calculated in cryoSPARC. (E) Local representative cryo-EM density map.

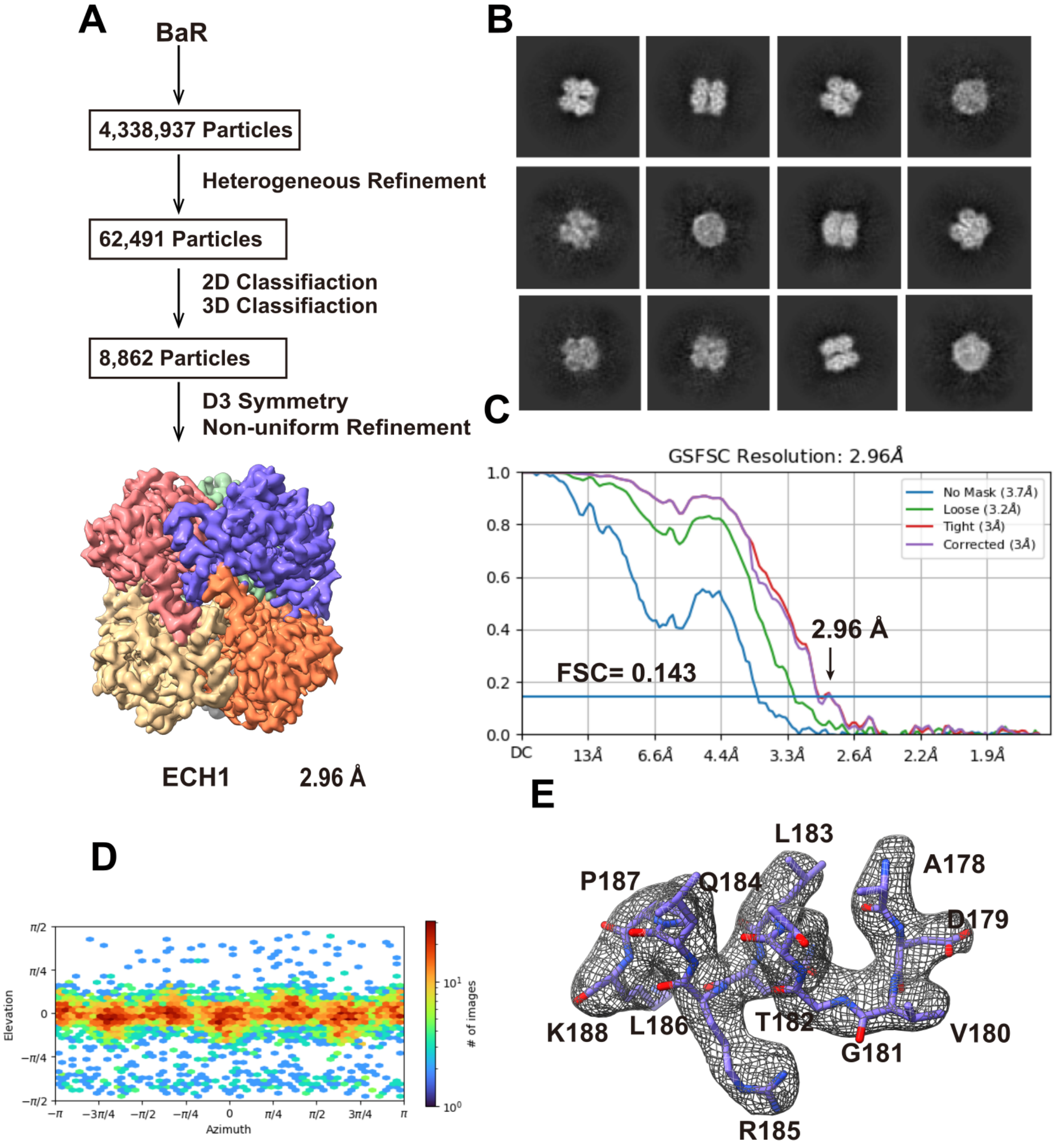

Figure S8. Cryo-EM structural determination of ECH1. (A) Particle stack workflow. (B) Representative 2D classes. (C) Fourier shell correlation curves, cutoff 0.143. (D) Angular distribution calculated in cryoSPARC. (E) Local representative cryo-EM density map.

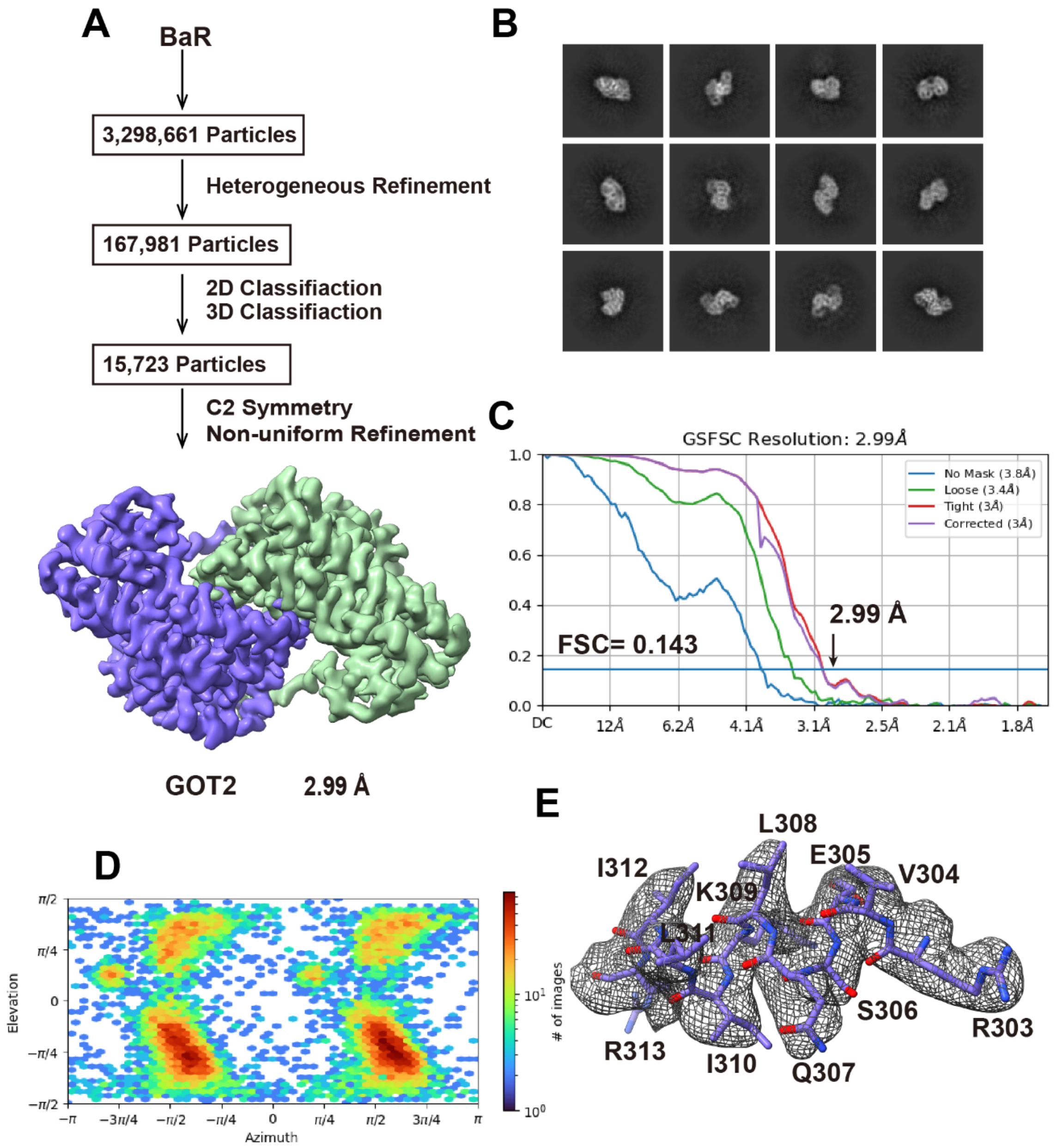

Figure S9. Cryo-EM structural determination of GOT2. (A) Particle stack workflow. (B) Representative 2D classes. (C) Fourier shell correlation curves, cutoff 0.143. (D) Angular distribution calculated in cryoSPARC. (E) Local representative cryo-EM density map.

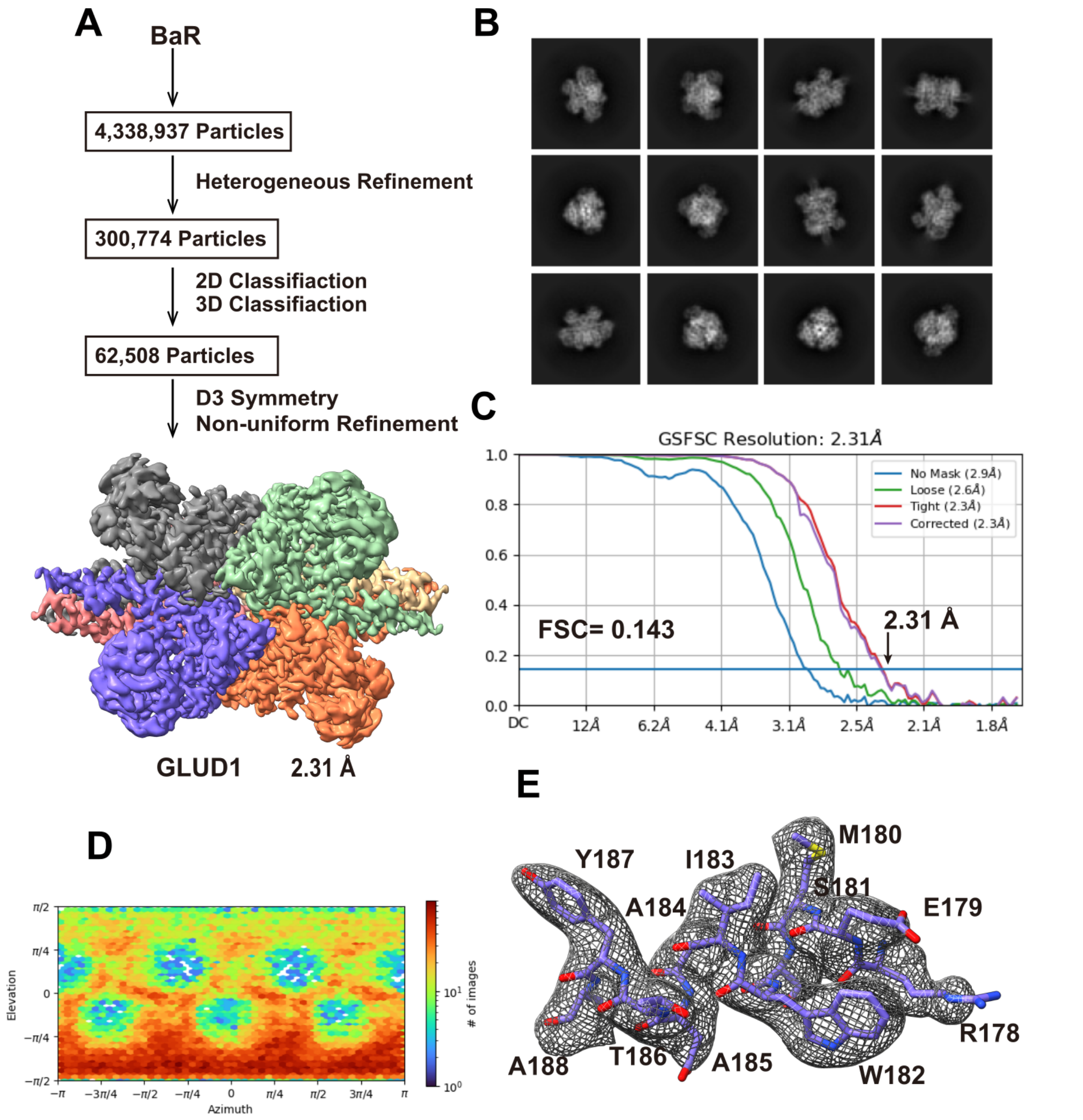

Figure S10. Cryo-EM structural determination of GLUD1. (A) Particle stack workflow. (B) Representative 2D classes. (C) Fourier shell correlation curves, cutoff 0.143. (D) Angular distribution calculated in cryoSPARC. (E) Local representative cryo-EM density map.

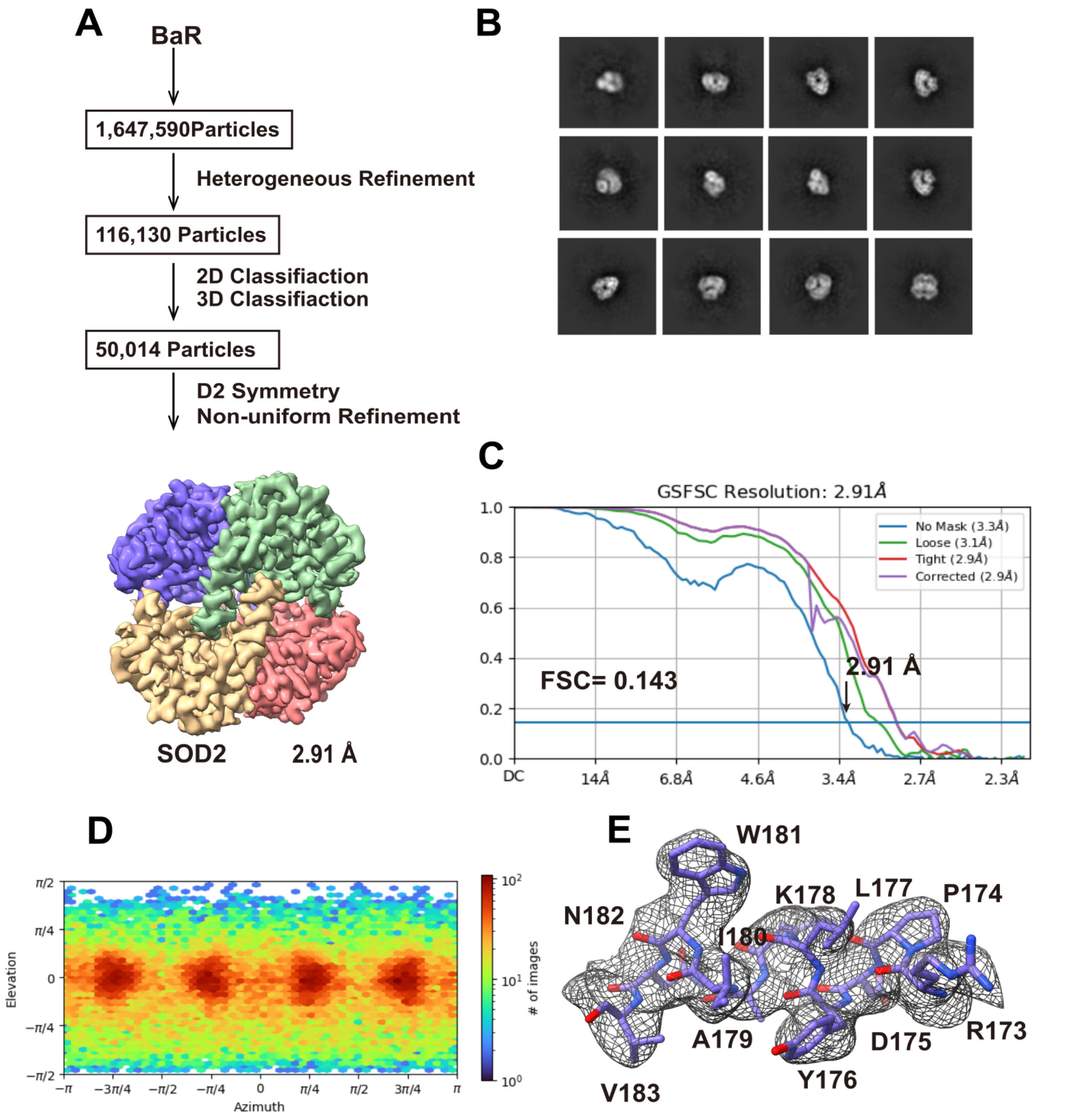

Figure S11. Cryo-EM structural determination of SOD2. (A) Particle stack workflow. (B) Representative 2D classes. (C) Fourier shell correlation curves, cutoff 0.143. (D) Angular distribution calculated in cryoSPARC. (E) Local representative cryo-EM density map.

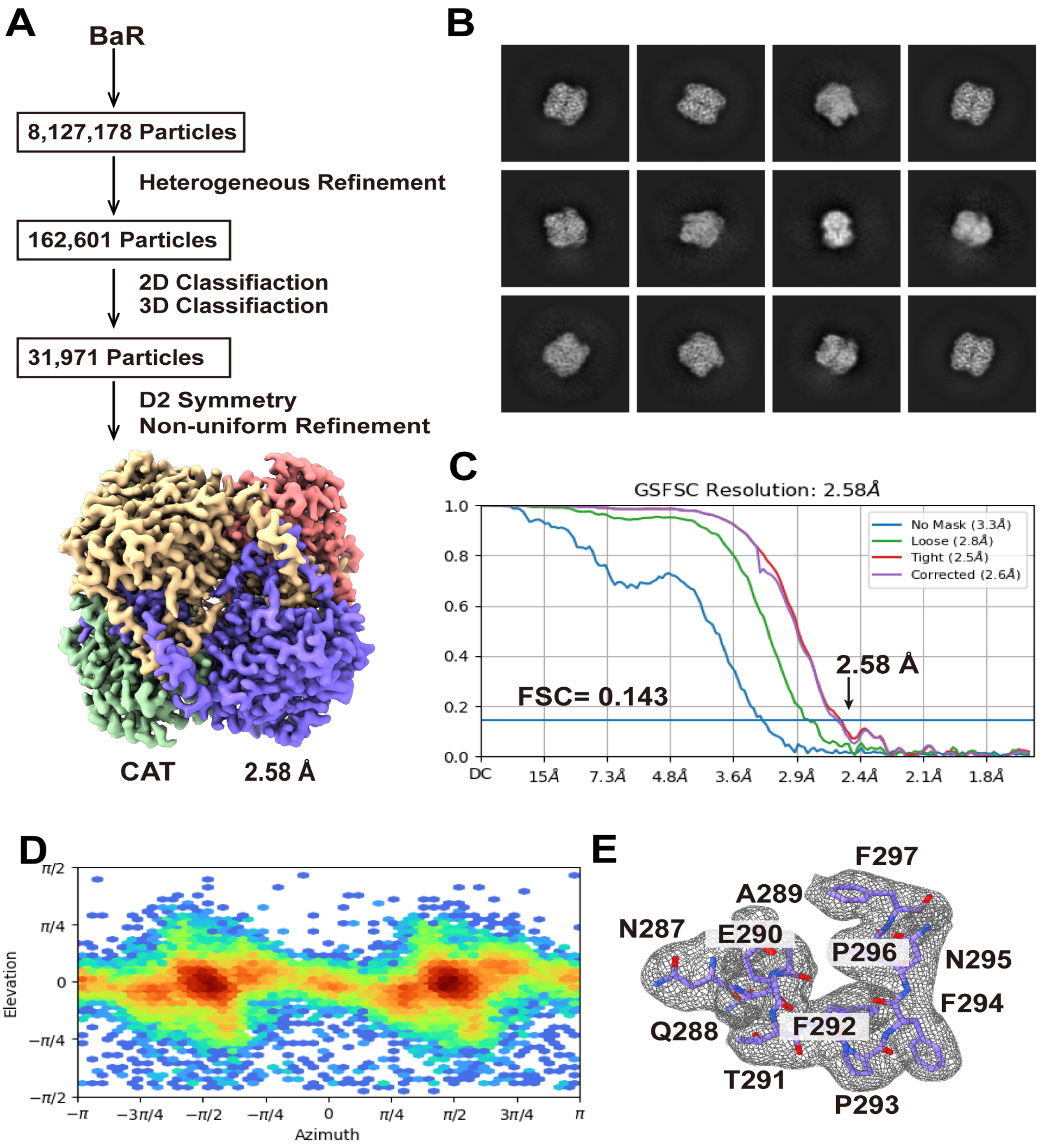

Figure S12. Cryo-EM structural determination of CAT. (A) Particle stack workflow. (B) Representative 2D classes. (C) Fourier shell correlation curves, cutoff 0.143. (D) Angular distribution calculated in cryoSPARC. (E) Local representative cryo-EM density map.

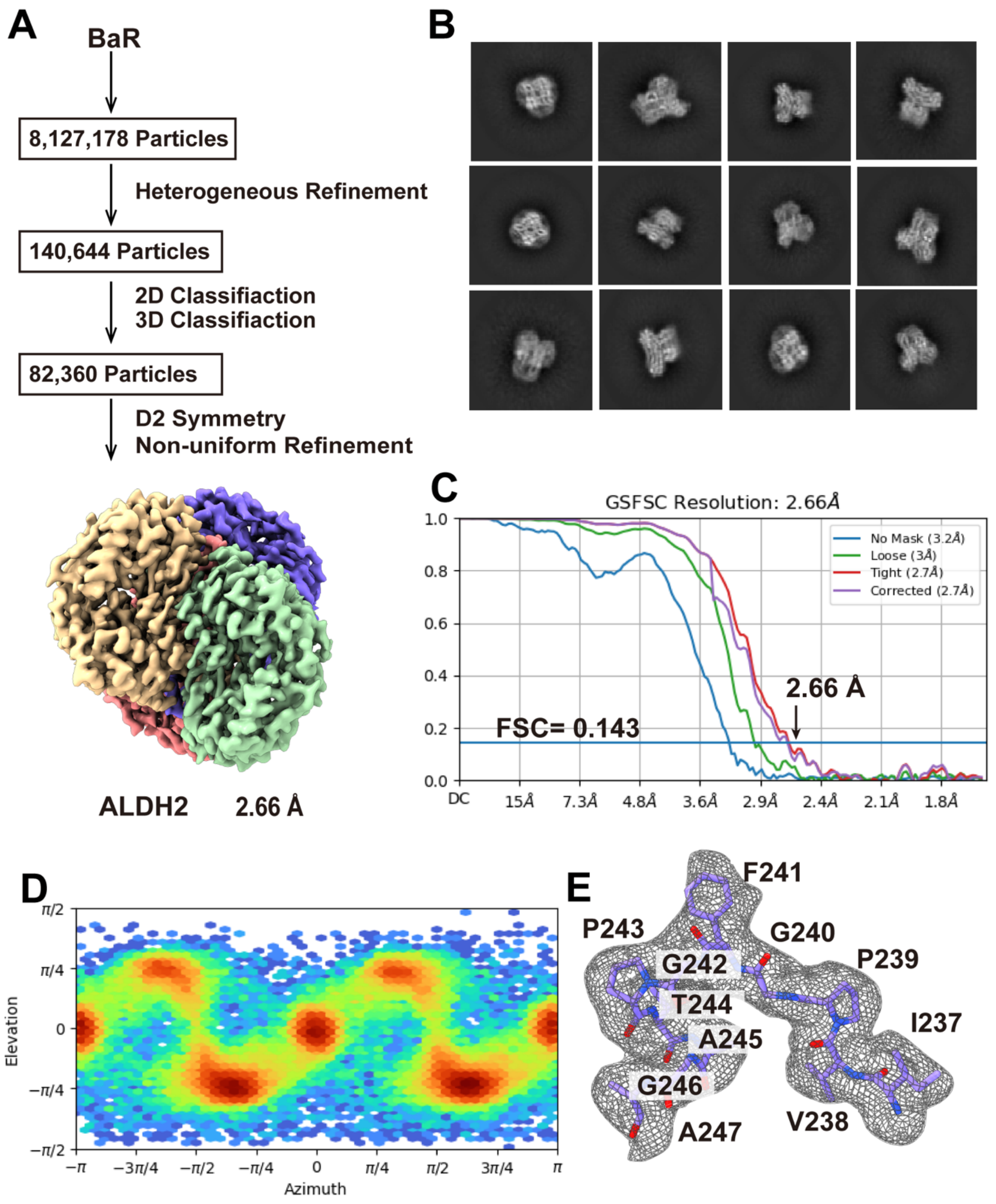

Figure S13. Cryo-EM structural determination of ALDH2. (A) Particle stack workflow. (B) Representative 2D classes. (C) Fourier shell correlation curves, cutoff 0.143. (D) Angular distribution calculated in cryoSPARC. (E) Local representative cryo-EM density map.

Table S1. Mitochondrial cryo-EM data collection and refinement statistics.

**Data collection**	**Peak 1 (200-450 kDa)**		**Peak 2 (150-250 kDa)**					**Peak 3 (80-150 kDa)**	
Magnification	105,000		105,000					105,000	81,000
Voltage (kV)	300		300					300	300
Electron Microscope	Krios-GIF-K3		Krios-GIF-K3					Krios-GIF-K3	Krios-GIF-K3
Energy filter width (eV)	20		20					20	20
Pixel size (Å)	0.825 (0.4125)		0.825 (0.4125)					0.825 (0.4125)	1.07 (0.535)
Total dose (e$^-$/Å^2)	35		35					36	39.5
Number of frames	40		40					35	47
Number of micrographs	7,966		8,756					8,259	2,272
Initial particle images (no.)	4,338,937		8,127,178					3,298,661	1,647,590
**Refinement**	**GLUD1**	**ECH1**	**SCAD**	**MCAD**	**IVD**	**CAT**	**ALDH2**	**GOT2**	**SOD2**
Total Particles (no.)	62,508	8,862	16,677	18,616	13,185	31,971	82,360	15,723	50,014
GS-FSC Resolution (0.143, Å)[a]	2.31	2.96	3.15	2.69	2.84	2.58	2.66	2.99	2.91
Model composition									
Chains	6	6	4	4	4	4	4	2	4
Protein residues	2,976	1,686	1,524	1,540	1,572	1,993	1,976	804	792
r.m.s.d.									
Bond lengths (Å)	0.004	0.002	0.004	0.002	0.004	0.004	0.002	0.004	0.004
Bond angles (°)	0.469	0.440	0.560	0.420	0.537	0.600	0.416	0.571	0.551
**Validation**									
MolProbity score	1.27	1.32	1.41	1.29	1.32	1.38	1.26	1.47	1.59
Clash score	4.84	5.79	5.44	4.12	5.61	3.69	5.02	7.13	7.37
Ramachandran plot									
Favored (%)	97.91	99.64	98.94	98.17	97.95	96.57	98.78	97.62	96.94
Allowed (%)	2.09	0.36	1.06	1.83	2.05	3.43	1.22	2.38	3.06
Disallowed (%)	0.00	0.00	0.00	0.00	0.00	0.00	0.00	0.00	0.00
CC Mask	0.89	0.88	0.81	0.80	0.78	0.86	0.81	0.85	0.84

[a]Gold-Standard Fourier-Shell Correlation

Table S2 Mitochondrial proteomic analysis

Rank	Log2 intensity	Log2 iBAQ[a]	Unique peptide	Mol. weight [kDa]	Score	MS/MS count	Sequence coverage [%]	Protein name and ID
**A. mitochondrial proteins (Peak 1: 200-450 kDa).**								
**1**	**36.60**	**30.59**	**71**	**61.397**	**323.31**	**847**	**74.9**	**P00367 Glutamate dehydrogenase 1**
2	36.37	30.36	79	61.054	323.31	1205	90.2	P10809 60 kDa heat shock protein
3	34.73	28.34	64	53.651	323.31	314	79.8	P08670 Vimentin
**4**	**32.69**	**27.83**	**34**	**35.816**	**323.31**	**429**	**88.1**	**Q13011 Delta(3,5)-Delta(2,4)-dienoyl-CoA isomerase**
5	30.32	27.83	7	11.514	67.549	42	99.1	P05386 60S acidic ribosomal protein P1
6	32.71	27.31	39	48.057	323.31	144	75.1	P05783 Keratin, type I cytoskeletal 18
7	31.97	27.10	15	27.692	323.31	140	50	P30048 Thioredoxin-dependent peroxide reductase
8	31.00	27.01	17	15.998	120.04	91	96.6	P68871 Hemoglobin subunit beta
9	32.91	26.88	54	55.637	323.31	173	71.5	Q3BDU5 Prelamin-A
10	33.14	26.84	51	72.25	323.31	378	72	D6RCD7 Stress-70 protein
**B. mitochondrial proteins (Peak 2: 150-250 kDa).**								
1	36.46	31.76	33	31.387	323.31	684	81	P30084 Enoyl-CoA hydratase
**2**	**37.06**	**31.05**	**55**	**56.381**	**323.31**	**739**	**87.6**	**P05091 Aldehyde dehydrogenase**
3	37.18	30.80	47	41.924	323.31	1151	97.5	P42765 3-ketoacyl-CoA thiolase
4	34.34	29.26	34	35.816	323.31	429	88.1	Q13011 Delta(3,5)-Delta(2,4)-dienoyl-CoA isomerase
5	37.04	29.24	156	164.94	323.31	1386	81.7	P31327 Carbamoyl-phosphate synthase [ammonia]
**6**	**34.87**	**29.19**	**41**	**44.297**	**323.31**	**347**	**81.1**	**P16219 Short-chain specific acyl-CoA dehydrogenase**
7	33.98	28.70	31	35.329	323.31	269	83.9	P31937 3-hydroxyisobutyrate dehydrogenase
8	34.98	28.67	6	45.199	323.31	442	85.7	P24752 Acetyl-CoA acetyltransferase
**9**	**33.96**	**28.66**	**36**	**46.588**	**323.31**	**233**	**68.2**	**P11310 Medium-chain specific acyl-CoA dehydrogenase**
10	34.52	28.65	32	39.473	323.31	203	85.4	P05062 Fructose-bisphosphate aldolase B
:								
**12**	**35.33**	**28.61**	**53**	**59.755**	**323.31**	**347**	**78.2**	**P04040 Catalase**
:								
**15**	**34.09**	**28.03**	**27**	**46.65**	**323.31**	**124**	**82.6**	**P26440 Isovaleryl-CoA dehydrogenase**
**C. mitochondrial proteins (Peak 3: 80-150 kDa).**								
1	35.92	30.79	41	35.503	323.31	314	74.9	P40926 Malate dehydrogenase
2	36.26	30.08	65	70.698	323.31	459	78.3	Q16822 Phosphoenolpyruvate carboxykinase [GTP]

3	35.50	30.07	11	34.293	323.31	344	87.3	Q16836 Hydroxyacyl-coenzyme A dehydrogenase
4	35.64	29.90	49	46.302	323.31	375	83.1	P50440 Glycine amidinotransferase
5	34.73	29.86	29	25.913	323.31	188	95.5	Q7Z4W1 L-xylulose reductase
**6**	**34.48**	**29.86**	**21**	**24.75**	**323.31**	**193**	**82.4**	**P04179 Superoxide dismutase [Mn]**
7	34.24	29.53	37	27.843	323.31	156	87.1	P38117 Electron transfer flavoprotein subunit beta
**8**	**35.18**	**29.26**	**43**	**47.517**	**323.31**	**237**	**75.6**	**P00505 Aspartate aminotransferase**
9	33.11	29.18	19	10.932	134.96	107	99	P61604 10 kDa heat shock protein
10	34.56	29.07	26	35.079	323.31	154	77.2	P13804 Electron transfer flavoprotein subunit alpha

[a]The iBAQ value is obtained by dividing protein intensities by the number of theoretically observable tryptic peptides between 6 and 30 amino acids, and is on average highly correlated with protein abundance.
